# Unified Entrainment and Detrainment Closures for Extended Eddy‐Diffusivity Mass‐Flux Schemes

**DOI:** 10.1029/2020MS002162

**Published:** 2020-09-14

**Authors:** Yair Cohen, Ignacio Lopez‐Gomez, Anna Jaruga, Jia He, Colleen M. Kaul, Tapio Schneider

**Affiliations:** ^1^ Department of Environmental Science and Engineering California Institute of Technology Pasadena CA USA; ^2^ Jet Propulsion Laboratory California Institute of Technology Pasadena CA USA; ^3^ Pacific Northwest National Laboratory Richland WA USA

**Keywords:** unified parameterization, convective parameterization, eddy‐diffusivity mass‐flux parameterization, entrainment and detrainment, deep convection, climate modeling

## Abstract

We demonstrate that an extended eddy‐diffusivity mass‐flux (EDMF) scheme can be used as a unified parameterization of subgrid‐scale turbulence and convection across a range of dynamical regimes, from dry convective boundary layers, through shallow convection, to deep convection. Central to achieving this unified representation of subgrid‐scale motions are entrainment and detrainment closures. We model entrainment and detrainment rates as a combination of turbulent and dynamical processes. Turbulent entrainment/detrainment is represented as downgradient diffusion between plumes and their environment. Dynamical entrainment/detrainment is proportional to a ratio of a relative buoyancy of a plume and a vertical velocity scale, that is modulated by heuristic nondimensional functions which represent their relative magnitudes and the enhanced detrainment due to evaporation from clouds in drier environment. We first evaluate the closures off‐line against entrainment and detrainment rates diagnosed from large eddy simulations (LESs) in which tracers are used to identify plumes, their turbulent environment, and mass and tracer exchanges between them. The LES are of canonical test cases of a dry convective boundary layer, shallow convection, and deep convection, thus spanning a broad rangeof regimes. We then compare the LES with the full EDMF scheme, including the new closures, in a single‐column model (SCM). The results show good agreement between the SCM and LES in quantities that are key for climate models, including thermodynamic profiles, cloud liquid water profiles, and profiles of higher moments of turbulent statistics. The SCM also captures well the diurnal cycle of convection and the onset of precipitation.

## Key Points

- An extended eddy‐diffusivity mass‐flux (EDMF) scheme successfully captures diverse regimes of convective motions
- Unified closures are presented for entrainment and detrainment across the different convective regimes
- With the unified closures, the EDMF scheme can simulate dry convection, shallow cumulus, and deep cumulus

## 1 Introduction

Turbulence and convection play an important role in the climate system. They transport energy, moisture, and momentum vertically, thereby controlling the formation of clouds and, especially in the tropics, the thermal stratification of the atmosphere. They occur on a wide range of scales, from motions on scales of meters to tens of meters in stable boundary layers and near the trade inversion, to motions on scales of kilometers in deep convection. General circulation models (GCMs), with horizontal resolutions approaching tens of kilometers, are unable to resolve this spectrum of motions. Turbulence and convection will remain unresolvable in GCMs for the foreseeable future (Schneider et al., 2017), although some deep convective motions, on scales of kilometers to tens of kilometers, are beginning to be resolved in short‐term global simulations (Kajikawa et al., 2016; Stevens et al., 2019).

Unable to resolve turbulence and convection explicitly, GCMs rely on parameterization schemes to represent subgrid‐scale (SGS) motions. Typically, GCMs have several distinct parameterization schemes for representing, for example, boundary layer turbulence, stratocumulus clouds, shallow convection, and deep convection. The different parameterization schemes interact via trigger functions with discontinuous behavior in parameter space, even though in reality the flow regimes they represent lie on a continuous spectrum (Xie et al., 2019). This fragmentary representation of SGS motion by multiple schemes leads to a proliferation of adjustable parameters, including parametric triggering functions that switch between schemes. Moreover, most existing parameterizations rely on statistical equilibrium assumptions between the SGS motions and the resolved scales. These assumptions become invalid as model resolution increases and, for example, some aspects of deep convection begin to be explicitly resolved (Dirmeyer et al., 2012; Gao et al., 2017). It is widely recognized that these issues make model calibration challenging and compromise our ability to make reliable climate predictions (Hourdin et al., 2017; Schmidt et al., 2017; Schneider et al., 2017).

Many known biases in climate models and uncertainties in climate predictions are attributed to difficulties in representing SGS turbulence and convection. For example, biases in the diurnal cycle and the continental near‐surface temperature, especially in polar regions, have been traced to inadequacies in turbulence parameterizations for stable boundary layers (Holtslag et al., 2013). Across climate models, biases in how tropical cloud cover covaries with temperature and other environmental factors on seasonal and interannual time scales are correlated with the equilibrium climate sensitivity, thus revealing the important role the representation of tropical low clouds plays in uncertainties in climate predictions (Bony & Dufresne, 2005; Brient & Schneider, 2016; Brient et al., 2016; Caldwell et al., 2018; Ceppi et al., 2017; Cesana et al., 2018; Dong et al., 2019; Lin et al., 2014; Nam et al., 2012; Schneider et al., 2019; Teixeira et al., 2011). Differences in moisture export from the mixed layer to the free troposphere by cumulus convection lead to differences in the width and strength of the ascending branch of the Hadley circulation (Neggers et al., 2007). And biases in the structure of the South Pacific Convergence Zone have been traced to biases in the intensity of deep convective updrafts (Hirota et al., 2014). It is evident from these few examples that progress in the representation of SGS turbulence and convection is crucial for progress in climate modeling and prediction. At the same time, it is desirable to unify the representation of SGS motions in one continuous parameterization scheme, to reduce the number of adjustable parameters and obtain a scheme that more faithfully represents the underlying continuum of physical processes.

Different approaches for a systematic coarse graining of the equations of motion, leading to a unified parameterization, have been proposed (de Rooy & Siebesma, 2010; Han & Bretherton, 2019; Lappen & Randall, 2001a; Park, 2014a, 2014b; Rio et al., 2019; Suselj et al., 2019b; Tan et al., 2018; Thuburn et al., 2018; Yano, 2014). They typically entail a conditional averaging (or filtering) of the governing equations over severalsubdomains (Weller & McIntyre, 2019), or an assumed probability density function (PDF) ansatz for dynamical variables and generation of moment equations from the ansatz (Golaz et al., 2002; Lappen & Randall, 2001a; Larson & Golaz, 2005; Larson et al., 2012). For example, conditional averaging can lead to a partitioning of a GCM grid box into subdomains representing coherent ascending and descending plumes, or drafts, and a more isotropically turbulent environment. Unclosed terms arise that, for example, to representinteractions among subdomains through entrainment and detrainment. Such unclosed terms need to be specified through closure assumptions (de Rooy et al., 2013). Or, if moment equations are generated through an assumed PDF ansatz for dynamical and thermodynamic variables, unclosed interactions among moments and dissipation terms need to be specified through closure assumptions (Golaz et al., 2002; Lappen & Randall, 2001b). Our goal in this paper is to develop a unified set of closures that work across the range of turbulent and convective motions, within one specific type of parameterization scheme known as an eddy‐diffusivity mass‐flux (EDMF) scheme (Siebesma & Teixeira, 2000; Siebesma et al., 2007; Wu et al., 2020).

We build on the extended EDMF scheme of Tan et al. (2018), which extends the original EDMF scheme of Siebesma and Teixeira (2000) by retaining explicit time dependence (SGS memory) and treating subdomain second‐moment equations consistently, so that, for example, energy exchange between plumes and their environment obeys conservation requirements. The explicit SGS memory avoids any statistical equilibrium assumption. This is a necessary ingredient for the scheme to become scale aware and be able to operate in the convective gray zone, where deep convective motions begin to become resolved.

In this and the companion paper Lopez‐Gomez et al. (2020) we present a set of unified closures that allow the extended EDMF parameterization to simulate stable boundary layers, dry convective boundary layers, stratocumulus‐topped boundary layers, shallow convection, and deep convection, all within a scheme with unified closures and a single set of parameters. This paper focuses on unified entrainment and detrainment closures that are essential for convective regime, and Lopez‐Gomez et al. (2020) present a closure for turbulent mixing. To demonstrate the viability of our approach, we compare the resulting parameterization scheme against large‐eddy simulations (LESs) of several canonical test cases for different dynamical regimes.

This paper is organized as follows. In section 2, we present the general structure of the extended EDMF scheme, including the subdomain decomposition and the prognostic equations for subdomain moments. Section 3 introduces the entrainment and detrainment closures that are key for the scheme to work across different dynamical regimes. Section 4 describes the numerical implementation of this scheme in a single‐column model (SCM). In section 5, we describe the LES used in this study and how we compare terms in the EDMF scheme against statistics derived from the LES. Section 6 compares results from the EDMF scheme against LES of canonical test cases of dry convective boundary layers, shallow, and deep convection. Section 7 summarizes and discusses the main findings.

## 2 Extended EDMF Scheme

### 2.1 Equations of Motion

The extended EDMF scheme is derived from the compressible equations of motion of the host model. As thermodynamic variables, we choose the liquid‐ice potential temperature *θ*_*l*_ and the total water specific humidity *q*_*t*_, but these choices can easily be modified and harmonized with the thermodynamic variables of the host model in which the scheme is implemented. The unfiltered governing equations are as follows:

(1) $$\frac{\partial \rho }{\partial t}+\nabla_{h}\cdot (\rho u_{h})+\frac{\partial (\rho w)}{\partial z}\;=0,$$

(2) $$\frac{\;\partial (\rho u_{h})}{\partial t}+\nabla_{h}\cdot (\rho u_{h}\;⊗\;u_{h})+\frac{\partial (\rho wu_{h})}{\partial z}\;=-\nabla_{h}p^{†}+\rho S_{u_{h}},$$

(3) $$\frac{\;\partial (\rho w)}{\partial t}+\nabla_{h}\cdot (\rho u_{h}w)+\;\frac{\partial (\rho ww)}{\partial z}\;=\rho b-\frac{\partial p^{†}}{\partial z}+\rho S_{w},$$

(4) $$\frac{\partial (\rho \theta_{l})}{\partial t}+\nabla_{h}\cdot (\rho u_{h}\theta_{l})+\frac{\partial (\rho w\theta_{l})}{\partial z}\;=\;\rho S_{\theta_{l}},$$

(5) $$\frac{\partial (\rho q_{t})}{\partial t}+\nabla_{h}\cdot (\rho u_{h}q_{t})+\frac{\partial (\rho wq_{t})}{\partial z}=\rho S_{q_{t}},$$

(6) $$p=\rho R_{d}T_{v}.$$

In the momentum equation, to improve numerical stability, we have removed a reference pressure profile *p*_*h*_(*z*) in hydrostatic balance with a density *ρ*_*h*_(*z*):

$$\frac{\partial p_{h}}{\partial z}=-\rho_{h}g,$$

where *g* is the gravitational acceleration. Therefore, the perturbation pressure

$$p^{†}=p-p_{h}$$

and the buoyancy

$$b=-\;g\;\frac{\rho -\rho_{h}}{\rho }$$

appear in the momentum equations in place of the full pressure *p* and gravitational acceleration *g*. Otherwise, the notation is standard: *ρ* is density, *q*_*t*_ is the total water specific humidity, *T*_*v*_ is the virtual temperature, *R*_*d*_ is the gas constant for dry air, and

(7) $$\theta_{l}=T{\left(\frac{p_{s}}{p}\right)}^{R_{d}/c_{p}}\exp\left(\frac{-L_{v}(q_{l}+q_{i})}{c_{p}T}\right)$$

is the liquid‐ice potential temperature, with liquid and ice specific humidities *q*_*l*_ and *q*_*i*_ and reference surface pressure *p*_*s*_ = 10^5^ Pa. In a common approximation that can easily be relaxed, we take the isobaric specific heat capacity of moist air *c*_*p*_ to be constant and, consistent with Kirchhoff's law, the latent heat of vaporization *L*_*v*_ to be a linear function of temperature (Romps, 2008). The temperature *T* is obtained from the thermodynamic variables *θ*_*l*_, *ρ*, and *q*_*t*_ by a saturation adjustment procedure, and the virtual temperature *T*_*v*_ is computed from the temperature *T* and the specific humidities (Pressel et al., 2015). The horizontal velocity vector is **u**_*h*_, and *w* is the vertical velocity component; *∇*_*h*_ is the horizontal nabla operator. The symbol *S* stands for sources and sinks. For the velocities, the sources $S_{u_{h}}$ and *S*_*w*_ include the molecular viscous stress and Coriolis forces, and for thermodynamic variables, the sources $S_{\theta_{l}}$ and $S_{q_{t}}$ represent sources from molecular diffusivity, microphysics, and radiation.

When implemented in a GCM, the host model solves for the grid‐averaged form of Equations 1–(6). In the averaged equations, SGS fluxes arise from the application of Reynolds averaging to quadratic and higher‐order terms. As is common, we make the boundary layer approximation and focus on the vertical SGS fluxes, neglecting horizontal SGS fluxes. The role of the parameterization in the host model is to predict these vertical SGS fluxes, in addition to cloud properties that are used by radiation and microphysics schemes. In the next section, a decomposition of grid boxes into subdomains expresses the vertical SGS fluxes as a sum of turbulent fluxes in the environment (ED) and convective mass fluxes in plumes (MF). To compute the MF component of the fluxes, the EDMF scheme solves for first moments of the host model's prognostic variables (*w*, *θ*_*l*_, *q*_*t*_) in each of its subdomains, as well as for the area fraction of the subdomains. To compute the ED component, the EDMF scheme solves additionally for the turbulence kinetic energy in the environment. Finally, to compute cloud properties by sampling from implied SGS distributions of thermodynamic variables, the EDMF scheme also solves for variances and covariance of *θ*_*l*_ and *q*_*t*_ in the environment. A summary of the prognostic and diagnostic variables in the scheme is given in Table 1.

**Table 1:** EDMF Scheme Variables

Symbol	Description	Unit	Prognostic	Diagnostic
$\rho ,{\bar{\rho }}_{i}$	Density	kg *m*^−*3*^		upd, env, gm
${\bar{p}}_{i},\langle p\rangle$	Pressure	Pa		upd, env, gm
*a*_*i*_	Subdomain area fraction		upd	env
${\bar{\theta }}_{l,i},\langle \theta_{l}\rangle$	Liquid‐ice potential temperature	K	upd, gm	env
${\bar{q}}_{t,i},\langle q_{t}\rangle$	Total water specific humidity	kg kg^−1^	upd, gm	env
${\bar{w}}_{i},\langle w\rangle$	Vertical velocity	m *s*^−*1*^	upd, gm	env
${\bar{u}}_{h,i}=\langle u_{h}\rangle$	Horizontal velocity	m *s*^−*1*^	gm	upd, env
${\bar{b}}_{i},\langle b\rangle$	Buoyancy	m s^−2^		env, upd, gm
$\bar{\theta_{l,0}^{\prime 2}},\langle \theta_{l}^{*2}\rangle$	*θ*_*l*_‐variance	*K*^*2*^	env	gm
$\bar{q_{t,0}^{\prime 2}},\langle q_{t}^{*2}\rangle$	*q*_*t*_‐variance	kg^2^ kg^−2^	env	gm
$\bar{\theta_{l,0}^{\prime }q_{t,0}^{\prime }},\langle \theta_{l}^{*}q_{t}^{*}\rangle$	Covariance of *θ*_*l*_ and *q*_*t*_	K kg kg^−1^	env	gm
${ē}_{0},\langle e\rangle$	Turbulence kinetic energy	m^2^ s^−2^	env	gm

*Note*. In the right two columns, “upd,” “env,” and “gm” stand for updrafts, environment, and grid mean, respectively, and these indicate whether a variable is prognostic or diagnostic in that model subdomain.

### 2.2 Domain Decomposition and Subdomain Moments

The extended EDMF scheme is derived from the equations of motion by decomposing the host model grid box into subdomains and averaging the equations over each subdomain volume. We denote by ⟨*ϕ*⟩ the average of a scalar *ϕ* over the host model grid box, with *ϕ*^∗^ = *ϕ* − ⟨*ϕ*⟩ denoting fluctuations about the grid mean. Similarly, ${\bar{\phi }}_{i}$ is the average of *ϕ* over the *i*th subdomain, and $\phi_{i}^{\prime }=\phi -{\bar{\phi }}_{i}$ is the fluctuation about the mean of subdomain *i*. The difference between the subdomain mean and grid mean then becomes ${\bar{\phi }}_{i}^{*}={\bar{\phi }}_{i}-\langle \phi \rangle$. Common terminology assigns an area fraction *a*_*i*_ = *A*_*i*_/*A*_*T*_ to each subdomain, where *A*_*i*_ is the horizontal area of the *i*th subdomain and *A*_*T*_ is the horizontal area of the grid box. This *a*_*i*_ is more precisely a volume fraction, since *A*_*i*_ is the vertically averaged horizontal area of the *i*th subdomain within the grid box. We retain here the terminology using subdomain area fractions, which reflect the subdomain volume fractions, consistent with previous works (Siebesma et al., 2007).

With this decomposition, the subdomain zeroth moment (area fraction), first moment (mean), centered second moment (covariance), and centered third moment obey:

(8) $$\underset{i\geq 0}{\sum }a_{i}\;=1,$$

(9) $$\langle \phi \rangle \;=\underset{i\geq 0}{\sum }a_{i}{\bar{\phi }}_{i},$$

(10) $$\begin{array}{ccc} \left\langle \phi^{*}\psi^{*}\right\rangle & \;=\underset{i\geq 0}{\sum }a_{i}\left[{\bar{\phi }}_{i}^{*}{\bar{\psi }}_{i}^{*}+\bar{\phi_{i}^{\prime }\psi_{i}^{\prime }}\right], & \\ & =\underset{i\geq 0}{\sum }\left[a_{i}\bar{\phi_{i}^{\prime }\psi_{i}^{\prime }}+\frac{1}{2}\underset{j\geq 0}{\sum }a_{i}a_{j}({\bar{\phi }}_{i}-{\bar{\phi }}_{j})({\bar{\psi }}_{i}-{\bar{\psi }}_{j})\right], \end{array}$$

(11) $$\begin{array}{cc} \left\langle \phi^{*}\psi^{*}w^{*}\right\rangle & =\underset{i\geq 0}{\sum }\left[a_{i}(\bar{\psi_{i}^{\prime }\phi_{i}^{\prime }w_{i}^{\prime }}+{\bar{\phi }}_{i}{\bar{\psi }}_{i}{\bar{w}}_{i}+{\bar{\psi }}_{i}\bar{w_{i}^{\prime }\phi_{i}^{\prime }}+{\bar{\phi }}_{i}\bar{w_{i}^{\prime }\psi_{i}^{\prime }}+{\bar{w}}_{i}\bar{\psi_{i}^{\prime }\phi_{i}^{\prime }})\right]\\ -\left[\left\langle \phi \rangle \langle \psi \rangle \langle w\rangle +\langle \phi \rangle \langle \psi^{*}w^{*}\rangle +\langle \psi \rangle \langle \phi^{*}w^{*}\rangle +\langle w\rangle \langle \psi^{*}\phi^{*}\right\rangle \right]. \end{array}$$

Equations (8) and (9) are self‐evident; the derivation of (10) and (11) from (8) and (9) is given in Appendix A. Equation 10 with *ϕ* = *w* is the vertical SGS flux of a scalar *ψ*, which is one of the key predictands of any parameterization scheme: The divergence of this flux appears as a source in the equations for the resolved scales of the host model. The decomposition in (9)–(11) only applies in general if $\bar{\left(\cdot \right)}$ is a Favre average—an average weighted by the density that appears in the continuity equation. However, in the EDMF scheme we describe in what follows, we make the approximation of ignoring density variations across subdomains (except in buoyancy terms), so that Favre and volume averages coincide within a grid box.

The central assumption in EDMF schemes is that within‐subdomain covariances such as $\bar{\phi_{i}^{\prime }\psi_{i}^{\prime }}$ and higher moments are neglected in all subdomains except one distinguished subdomain, the environment, denoted by index *i* = 0. In the environment, covariances $\bar{\phi_{0}^{\prime }\psi_{0}^{\prime }}$ are retained, and third moments such as $\bar{w_{0}^{\prime }\phi_{0}^{\prime }\psi_{0}^{\prime }}$, which appear in second‐moment equations, are modeled with closures. The intuition underlying this assumption is that the flow domain is subdivided into an isotropically turbulent environment (*i* = 0) and into coherent structures, identified with plumes (*i* ≥ 1). The environment can have substantial within‐subdomain covariances, whereas the plumes are taken to have comparatively little variance within them. Variance within plumes can be represented by having an ensemble of plumes with different mean values (Neggers, 2012; Neggers et al., 2002; Sušelj et al., 2012). For the case of only two subdomains, an updraft (*i* = 1) and its environment (*i* = 0), the second‐moment Equation 10 then simplifies to

(12) $$\begin{array}{ccc} \left\langle \phi^{*}\psi^{*}\right\rangle & =a_{1}\bar{\phi_{1}^{\prime }\psi_{1}^{\prime }}+(1-a_{1})\bar{\phi_{0}^{\prime }\psi_{0}^{\prime }}+a_{1}(1-a_{1})({\bar{\phi }}_{1}-{\bar{\phi }}_{0})({\bar{\psi }}_{1}-{\bar{\psi }}_{0}) & \\ & \;\approx \underset{\text{ED}}{\underbrace{(1-a_{1})\bar{\phi_{0}^{\prime }\psi_{0}^{\prime }}}}+\underset{\text{MF}}{\underbrace{a_{1}(1-a_{1})({\bar{\phi }}_{1}-{\bar{\phi }}_{0})({\bar{\psi }}_{1}-{\bar{\psi }}_{0})}}, \end{array}$$

where the approximation in the second line reflects the EDMF assumption of neglecting within‐plume covariances. The first line states that the covariance on the grid scale can be decomposed into the sum of the covariances within subdomains and the covariance among subdomain means, as in the analysis of variance (ANOVA) from statistics (Mardia et al., 1979). The second line reflects the EDMF approximation to only retain the covariances in the environment. The first term on the right‐hand side is closed by a downgradient eddy diffusion (ED) closure and the second term is represented by a mass flux (MF) closure, whence EDMF derives its name (Siebesma & Teixeira, 2000). Whenever *ϕ* and *ψ* are both thermodynamic prognostic variables, the within‐environment covariance $\bar{\phi_{0}^{\prime }\psi_{0}^{\prime }}$ is solved prognostically. Under the EDMF assumption, the third‐moment Equation (11) for two subdomains, written for a single scalar, simplifies to

(13) $$\langle \phi^{*}\phi^{*}\phi^{*}\rangle \approx -a_{1}(1-a_{1})({\bar{\phi }}_{1}-{\bar{\phi }}_{0})\bar{\phi_{0}^{\prime }\phi_{0}^{\prime }}+3a_{1}(1-a_{1})(1-2a_{1}){\left({\bar{\phi }}_{1}-{\bar{\phi }}_{0}\right)}^{3}.$$

That is, third moments (i.e., skewness) on the grid scale are represented through covariances within the environment and through variations among means across subdomains with differing area fractions.

### 2.3 EDMF Assumptions

The extended EDMF scheme is obtained by applying this decomposition of grid‐scale variations to the equations of motion (1)–(6), making the following additional assumptions:

1. We make the boundary layer approximation for subgrid scales, meaning that we assume vertical derivatives to be much larger than horizontal derivatives. This in particular means that the diffusive closure for fluxes in the environment only involves vertical gradients,
  (14) $$\bar{w_{i}^{\prime }\phi_{i}^{\prime }}\approx -K_{\phi ,i}\frac{\partial {\bar{\phi }}_{i}}{\partial z},$$
  where *K*_*ϕ*,*i*_ is the eddy diffusivity (to be specified) for scalar *ϕ* in subdomain *i*. Consistent with the EDMF assumptions, we assume *K*_*ϕ*,*i*_ = 0 for *i* ≠ 0.
2. We use the same, grid‐mean density ⟨*ρ*⟩ in all subdomains except in the buoyancy term. This amounts to making an anelastic approximation on the subgrid scale, to suppress additional acoustic modes that would otherwise arise through the domain decomposition. For notational simplicity, we use *ρ* rather than ⟨*ρ*⟩ for the grid‐mean density in what follows, and ${\bar{\rho }}_{i}$ for the subdomain density that appears only in the buoyancy term:
  (15) $${\bar{b}}_{i}=-g\frac{{\bar{\rho }}_{i}-\rho_{h}}{\rho }.$$
  The grid‐mean density *ρ* appears in the denominator, playing the role of the reference density in the anelastic approximation. The area fraction‐weighted sum of the subdomain buoyancies is the grid‐mean buoyancy, ensuring consistency of this decomposition:
  (16) $$\langle b\rangle =\underset{i}{\sum }a_{i}{\bar{b}}_{i}=-g\frac{\rho -\rho_{h}}{\rho }.$$
3. We take the subdomain horizontal velocities to be equal to their grid‐mean values,
  (17) $${\bar{u}}_{h,i}=\langle u_{h}\rangle .$$
  This simplification is commonly made in parameterizations for climate models (Larson et al., 2019). It eliminates mass‐flux contributions to the SGS vertical flux of horizontal momentum.

### 2.4 EDMF Equations

The full derivation of the subdomain‐mean and covariance equations from (1)–(6) is given in Appendix B. The derivation largely follows Tan et al. (2018), except for a distinction between dynamical and turbulent entrainment and detrainment following de Rooy and Siebesma (2010). The resulting extended EDMF equation for the subdomain area fraction is

(18) $$\frac{\partial (\rho a_{i})}{\partial t}+\nabla_{h}\cdot (\rho a_{i}\langle u_{h}\rangle )+\frac{\partial (\rho a_{i}{\bar{w}}_{i})}{\partial z}=\underset{j\neq i}{\sum }\left(E_{ij}-\Delta_{ij}\right);$$

the equation for the subdomain‐mean vertical momentum is

(19) $$\begin{array}{ccc} \frac{\partial (\rho a_{i}{\bar{w}}_{i})}{\partial t}+\nabla_{h}\cdot (\rho a_{i}\langle u_{h}\rangle {\bar{w}}_{i}) & +\frac{\partial (\rho a_{i}{\bar{w}}_{i}{\bar{w}}_{i})}{\partial z}= & \\ \frac{\partial }{\partial z}\left(\rho a_{i}K_{w,i}\frac{\partial \bar{w_{i}}}{\partial z}\right) & +\underset{j\neq i}{\sum }\left[(E_{ij}+{Ê}_{ij}){\bar{w}}_{j}-(\Delta_{ij}+{Ê}_{ij}){\bar{w}}_{i}\right]\\ & +\rho a_{i}({\bar{b}}_{i}^{*}+\langle b\rangle )-\rho a_{i}\frac{\partial }{\partial z}\left(\frac{{\bar{p}}_{i}^{*}+\langle p^{†}\rangle }{\rho }\right)+{\bar{S}}_{w,i}; \end{array}$$

and the equation for the subdomain‐mean of a thermodynamic scalar *ϕ* is

(20) $$\begin{array}{ccc} \frac{\partial (\rho a_{i}{\bar{\phi }}_{i})}{\partial t}+\nabla_{h}\cdot (\rho a_{i}\langle u_{h}\rangle {\bar{\phi }}_{i}) & +\frac{\partial (\rho a_{i}{\bar{w}}_{i}{\bar{\phi }}_{i})}{\partial z}= & \\ \frac{\partial }{\partial z}\left(\rho a_{i}K_{\phi ,i}\frac{\partial \bar{\phi_{i}}}{\partial z}\right) & +\underset{j\neq i}{\sum }\left[(E_{ij}+{Ê}_{ij}){\bar{\phi }}_{j}-(\Delta_{ij}+{Ê}_{ij}){\bar{\phi }}_{i}\right]+\rho a_{i}{\bar{S}}_{\phi ,i}. \end{array}$$

The dynamical entrainment rate from subdomain *j* into subdomain *i* is *E*_*ij*_, and the detrainment rate from subdomain *i* into subdomain *j* is Δ_*ij*_. In addition to dynamical entrainment, there is turbulent entrainment from subdomain *j* into subdomain *i*, with rate ${Ê}_{ij}$. Turbulent entrainment differentially entrains tracers but not mass (see Appendix B).

The pressure and buoyancy terms in the vertical momentum Equation (19) are written as the sum of their grid‐mean value and perturbations from their grid‐mean value. These perturbations vanish when summed over all subdomains because $\sum_{i}a_{i}{\bar{\phi }}_{i}^{*}=0$; hence, the grid‐mean values of the pressure and buoyancy terms are recovered upon summing over subdomains. Following Pauluis (2008), the pressure gradient term in (19) is written with 1/*ρ* inside the gradient to ensure energy conservation in our SGS anelastic approximation; see Appendix C for details. The subdomain density ${\bar{\rho }}_{i}$ that is essential for the subdomain buoyancy is computed from the subdomain virtual temperature ${\bar{T}}_{v,i}$ using the ideal gas law with the grid‐mean pressure ⟨*p*⟩:

(21) $${\bar{\rho }}_{i}=\frac{\left\langle p\right\rangle }{R_{d}{\bar{T}}_{v,i}}.$$

In analogy with the anelastic approximation of Pauluis (2008), this formulation of the ideal gas law ensures that $\sum_{i}a_{i}{\bar{\rho }}_{i}{\bar{T}}_{v,i}=\rho \langle T_{v}\rangle$, while accounting for subdomain virtual temperature effects that play a key role in the buoyancy of updrafts in shallow convection.

The scalar Equation (20) is applied to any thermodynamic variable, with its corresponding subdomain‐averaged source ${\bar{S}}_{\phi ,i}$ on the right‐hand side. The terms on the left‐hand side represent the explicit time tendencies and fluxes of the subdomain‐means, which can be viewed as forming part of the dynamical core of the host model. The terms on the right‐hand side are sources and sinks that require closure. The covariance equation for thermodynamic scalars (i.e., when *ϕ*,*ψ* ∈ [*θ*_*l*_, *q*_*t*_]) in the environment becomes

(22) $$\begin{array}{ccc} \frac{\partial (\rho a_{0}\bar{\phi_{0}^{\prime }\psi_{0}^{\prime }})}{\partial t} & +\nabla_{h}\cdot (\rho a_{0}\langle u_{h}\rangle \bar{\phi_{0}^{\prime }\psi_{0}^{\prime }}))+\underset{\text{vertical transport}}{\underbrace{\frac{\partial (\rho a_{0}{\bar{w}}_{0}\bar{\phi_{0}^{\prime }\psi_{0}^{\prime }})}{\partial z}}}= & \\ & \;\underset{\text{turbulent transport}}{\underbrace{\frac{\partial }{\partial z}\left(\rho a_{0}K_{\phi \psi ,0}\frac{\partial \bar{\phi_{0}^{\prime }\psi_{0}^{\prime }}}{\partial z}\right)}}+\underset{\text{turbulent production}}{\underbrace{2\rho a_{0}K_{\phi \psi ,0}\frac{\partial \bar{\phi_{0}}}{\partial z}\frac{\partial \bar{\psi_{0}}}{\partial z}}}\\ & +\underset{i>0}{\sum }\left(\underset{\text{turb. entrainment}}{\underbrace{-{Ê}_{0i}\bar{\phi_{0}^{\prime }\psi_{0}^{\prime }}}}+\underset{\text{turb. entrainment production}}{\underbrace{{\bar{\psi }}_{0}^{*}{Ê}_{0i}({\bar{\phi }}_{0}-{\bar{\phi }}_{i})+{\bar{\phi }}_{0}^{*}{Ê}_{0i}({\bar{\psi }}_{0}-{\bar{\psi }}_{i})}}\right)\\ & +\underset{i>0}{\sum }\left(\underset{\text{dyn. detrainment}}{\underbrace{-\Delta_{0i}\bar{\phi_{0}^{\prime }\psi_{0}^{\prime }}}}+\underset{\text{dyn. entrainment flux}}{\underbrace{E_{0i}({\bar{\phi }}_{0}-{\bar{\phi }}_{i})({\bar{\psi }}_{0}-{\bar{\psi }}_{i})}}\right)\\ & -\underset{\text{dissipation}}{\underbrace{\rho a_{0}\bar{D_{\phi^{\prime }\psi^{\prime },0}}}}+\rho a_{0}(\bar{\psi_{0}^{\prime }S_{\phi ,0}^{\prime }}+\bar{\phi_{0}^{\prime }S_{\psi ,0}^{\prime }}). \end{array}$$

Consistently with the EDMF assumption, we have assumed here that $\bar{\phi_{i}^{\prime }\psi_{i}^{\prime }}=0$ for *i* > 0. Covariance equations of this form are used for the thermodynamic variances $\bar{\theta_{l,0}^{\prime 2}}$ and $\bar{q_{t,0}^{\prime 2}}$ and for the covariance $\bar{\theta_{l,0}^{\prime }q_{t,0}^{\prime }}$, which are needed in microphysics parameterizations. Note that some of the entrainment and detrainment terms are cross‐subdomain counterparts of the vertical gradient terms. For example, the “dynamical entrainment,” “turbulent entrainment,” and “turbulent entrainment production” are the cross‐subdomain counterparts of the “vertical transport,” “turbulent transport,” and “turbulent production,” respectively. The “dynamical entrainment flux” lacks any vertical counterpart. This term arises as a flux across a variable boundary in the conditional averaging process.

The subdomain turbulence kinetic energy (TKE) is defined as ${ē}_{i}=0.5(\bar{u_{i}^{\prime 2}}+\bar{v_{i}^{\prime 2}}+\bar{w_{i}^{\prime 2}})$, and the TKE equation for the environment is written as

(23) $$\begin{array}{ccc} \frac{\partial (\rho a_{0}{ē}_{0})}{\partial t}+\nabla_{h}\cdot (\rho a_{0}\langle u_{h}\rangle {ē}_{0}) & \frac{\partial (\rho a_{0}{\bar{w}}_{0}{ē}_{0})}{\partial z}= & \\ \underset{\text{turbulent transport}}{\underbrace{\frac{\partial }{\partial z}\left(\rho a_{0}K_{m,0}\frac{\partial {ē}_{0}}{\partial z}\right)}} & +\underset{\text{shear production}}{\underbrace{\rho a_{0}K_{m,0}\left[{\left(\frac{\partial \langle u\rangle }{\partial z}\right)}^{2}+{\left(\frac{\partial \langle v\rangle }{\partial z}\right)}^{2}+{\left(\frac{\partial {\bar{w}}_{0}}{\partial z}\right)}^{2}\right]}}\\ & +\underset{i>0}{\sum }\left(\underset{\text{turb. entrainment}}{\underbrace{-{Ê}_{0i}{ē}_{0}}}+\underset{\text{turb. entrainment production}}{\underbrace{{\bar{w}}_{0}^{*}{Ê}_{0i}({\bar{w}}_{0}-{\bar{w}}_{i})}}\right)\\ & +\underset{i>0}{\sum }\left(\underset{\text{dyn. detrainment}}{\underbrace{-\Delta_{0i}{ē}_{0}}}+\frac{1}{2}\;\underset{\text{dyn. entrainment production}}{\underbrace{E_{0i}({\bar{w}}_{0}-{\bar{w}}_{i})({\bar{w}}_{0}-{\bar{w}}_{i})}}\right)\\ +\underset{\text{buoyancy production}}{\underbrace{\rho a_{0}\bar{w_{0}^{\prime }b_{0}^{\prime }}}} & -\underset{\text{pressure term}}{\underbrace{\rho a_{0}\left[\bar{u_{0}^{\prime }\frac{\partial }{\partial x}{\left(\frac{p^{†}}{\rho }\right)}_{0}^{\prime }}+\bar{v_{0}^{\prime }\frac{\partial }{\partial y}{\left(\frac{p^{†}}{\rho }\right)}_{0}^{\prime }}+\bar{w_{0}^{\prime }\frac{\partial }{\partial z}{\left(\frac{p^{†}}{\rho }\right)}_{0}^{\prime }}\right]}}-\underset{\text{dissipation}}{\underbrace{\rho a_{0}\bar{D_{e,0}}};} \end{array}$$

see Appendix B in Lopez‐Gomez et al. (2020) for a detailed derivation of the TKE equation. We have used the EDMF assumption that ${ē}_{i}\approx 0$ for *i* > 0. The prognostic TKE is used for closures of the eddy diffusivity in the environment as described in Lopez‐Gomez et al. (2020).

### 2.5 Effect on Grid Mean and Constraints on Entrainment/Detrainment

The conservation of mass and scalars in the host model grid box requires that by summing the EDMF equations over all subdomains, the equations for the grid‐mean variables are recovered. The horizontal flux divergence terms that are included in the EDMF equations, $\nabla_{h}\cdot (\rho a_{i}\langle u_{h}\rangle {\bar{\phi }}_{i})$, represent the fluxes across the boundaries of the host model grid (see Appendix B) and, when summed over all subdomains, recover their grid‐mean counterpart. Additionally, mass conservation requires that between two subdomains *i* and *j*, the entrainment and detrainment rates satisfy (*E*_*ij*_ − Δ_*ij*_) + (*E*_*ji*_ − Δ_*ji*_) = 0. For entrainment and detrainment of subdomain‐mean properties, scalar conservation further requires that

(24) $$E_{ij}=\Delta_{ji},$$

so that when summing over two interacting subdomains, the entrainment and detrainment terms cancel out. Similarly, scalar conservation requires symmetry, ${Ê}_{ij}={Ê}_{ji}$, for turbulent entrainment.

Taking these requirements into account, a summation of Equation (20) over all subdomains yields the grid‐mean scalar equation

(25) $$\frac{\partial (\rho \langle \phi \rangle )}{\partial t}+\nabla_{h}\cdot (\rho \langle u_{h}\rangle \langle \phi \rangle )+\frac{\partial (\rho \langle w\rangle \langle \phi \rangle )}{\partial z}=-\frac{\partial }{\partial z}(\rho \langle w^{*}\phi^{*}\rangle )+\rho \langle S_{\phi }\rangle .$$

This is the form of the equation solved by the dynamical core of the host model. Using the covariance decomposition (10), the SGS flux in (25) is written as the sum of the eddy diffusivity and mass flux components:

(26) $$\rho \langle w^{*}\phi^{*}\rangle =-\rho a_{0}K_{\phi ,0}\frac{\partial {\bar{\phi }}_{0}}{\partial z}+\underset{i\geq 0}{\sum }\rho a_{i}({\bar{w}}_{i}-\langle w\rangle )({\bar{\phi }}_{i}-\langle \phi \rangle ).$$

This illustrates the coupling between the dynamical core equations and the EDMF scheme. Similarly, the grid covariance equation follows by using the subdomain continuity equation (18), scalar‐mean equation (20), and the scalar covariance equation (22) in the covariance decomposition (10), which yields

(27) $$\begin{array}{ccc} \frac{\partial (\rho \langle \phi^{*}\psi^{*}\rangle )}{\partial t} & +\nabla_{h}\cdot (\rho \langle u_{h}\rangle \langle \phi^{*}\psi^{*}\rangle )+\frac{\partial (\rho \langle w\rangle \langle \phi^{*}\psi^{*}\rangle )}{\partial z}= & \\ & -\frac{\partial (\rho \langle w^{*}\phi^{*}\psi^{*}\rangle )}{\partial z}-\rho \langle w^{*}\psi^{*}\rangle \frac{\partial \langle \phi \rangle }{\partial z}-\rho \langle w^{*}\phi^{*}\rangle \frac{\partial \langle \psi \rangle }{\partial z}\\ & -\rho \langle D_{\phi^{*}\psi^{*}}\rangle +\rho \langle \psi^{*}S_{\phi }^{*}\rangle +\rho \langle \phi^{*}S_{\psi }^{*}\rangle . \end{array}$$

Here, vertical SGS fluxes are decomposed according to Equation (26), and the turbulent transport term is decomposed according to Equation (11). In general, Equation (27) does not need to be solved by the host model. However, the consistency of the summation over subdomains to produce it ensures that the second moments are conserved within the EDMF scheme.

The subdomain equations in the EDMF scheme require closures for dynamical entrainment and detrainment, turbulent entrainment, perturbation pressure, eddy diffusivity, for the various sources, and for covariance dissipation. The following section focuses on closures for dynamical and turbulent entrainment and detrainment. The perturbation pressure closure is given by the sum of a virtual mass effect, momentum convergence, and pressure drag, see equation (11) in Lopez‐Gomez et al. (2020). The eddy diffusivity and mixing length closures are described in Lopez‐Gomez et al. (2020).

## 3 Closures

Entrainment and detrainment closures are a topic of extensive research (de Rooy et al., 2013). Following de Rooy and Siebesma (2010), we distinguish dynamical and turbulent entrainment and detrainment components. Turbulent entrainment is typically represented by a diffusive horizontal flux, while diverse closures for dynamical entrainment and detrainment are in use. It is common to write the dynamical entrainment and detrainment rates as a product of the vertical mass flux $\rho a_{i}{\bar{w}}_{i}$ and fractional entrainment/detrainment rates *ϵ*_*ij*_ and *δ*_*ij*_

(28) $$E_{ij}=\rho a_{i}{\bar{w}}_{i}\epsilon_{ij},$$

and

(29) $$\Delta_{ij}=\rho a_{i}{\bar{w}}_{i}\delta_{ij}.$$

Closures are then derived for the fractional rates *ϵ*_*ij*_ and *δ*_*ij*_ per unit length (they have units of 1/length).

Various functional forms for the fractional rates *ϵ*_*ij*_ and *δ*_*ij*_ have been proposed in the literature. For example,

- Based on experiments on dry thermals, Morton et al. (1956) suggested *ϵ*_*ij*_ to be inversely proportional to the updraft radius. This relation has been used in several closures (Bretherton et al., 2004; Kain & Fritsch, 1990).
- Using a perturbation response experiment in LES of shallow convection, Tian and Kuang (2016) found $\epsilon_{i0}\propto 1/({\bar{w}}_{i}\tau )$ with a mixing time scale *τ*. Such an entrainment rate was used by Neggers et al. (2002), Sušelj et al. (2012), and Langhans et al. (2019) in shallow convection parameterizations.
- Gregory (2001) analyzed LES of shallow convection and suggested $\epsilon_{i0}\propto {\bar{b}}_{i}/{\bar{w}}_{i}^{2}$, which was used by Tan et al. (2018) for shallow convection. The ratio ${\bar{w}}_{i}/{\bar{b}}_{i}$ plays the role of the time scale *τ* in the formulation of Tian and Kuang (2016). In the steady equations, this entrainment functional form also ensures that the mass flux and the vertical velocity simultaneously go to zero at the top of updrafts; see Appendix E and Romps (2016). Alternative derivations of this functional form are based on a balance of sources and sinks of total kinetic energy in updrafts (Savre & Herzog, 2019), or on the dynamics of dry thermals (McKim et al., 2020).
- Other approaches for entrainment and detrainment include stochastic closures (Romps, 2016; Suselj et al., 2013, 2014, 2019a) and higher‐order closures (Lappen & Randall, 2001b).

Similar closures are often used for both entrainment *ϵ*_*ij*_ and detrainment *δ*_*ij*_. Enhanced detrainment can occur in cloudy conditions: When the evaporation of cloud condensate after mixing with drier environmental air produces a buoyancy sink for an updraft, negatively buoyant air can detrain rapidly from the updraft (Kain & Fritsch, 1990; Raymond & Blyth, 1986). Various approaches for representing this enhanced detrainment owing to “buoyancy sorting” have been used, ranging from adding a constant background detrainment rate (Siebesma & Cuijpers, 1995; Tan et al., 2018), over explicitly modeling buoyancies of mixtures of cloudy and environmental air (Bretherton et al., 2004; Kain & Fritsch, 1990), to enhancing detrainment by functions of updraft‐environment relative humidity differences (Bechtold et al., 2008, 2014; Böing et al., 2012; Savre & Herzog, 2019).

Here we combine insights from several of these studies into a new closure for entrainment and detrainment.

### 3.1 Dynamical Entrainment and Detrainment

We propose closures for dynamical entrainment and detrainment that are in principle applicable to many interacting subdomains (e.g., multiple updrafts, or updrafts and downdrafts). Our point of departure are dry entrainment and detrainment rates which are symmetric for upward and downward motions. To those we then add the contribution of evaporation, which is asymmetric between upward and downward motions. We first write our closures for the rates *E*_*ij*_ and Δ_*ij*_, which facilitate ensuring mass and scalar conservation. In the end, we give the corresponding formulations in terms of the fractional rates *ϵ*_*ij*_ and *δ*_*ij*_.

#### 3.1.1 General Form of Entrainment and Detrainment Rates

The rates *E*_*ij*_ and Δ_*ij*_ have units of density divided by time and hence depend on a flow‐dependent time scale, as well as on functions of nondimensional groups in the problem. Following Gregory (2001), Tan et al. (2018), Savre and Herzog (2019), and McKim et al. (2020), among others, we choose an inverse time scale *b*/*w* as the fundamental scale, depending on a buoyancy *b* and a vertical velocity *w*. This vertical velocity scale is taken to be representative of the vertical velocity difference across the updraft boundary, which we approximate as the difference between the subdomain means in convective conditions. In cases of strong environmental turbulence and weak updraft velocities, the environmental turbulent velocity scale ${ē}_{0}^{1/2}$ is a better representation of this velocity difference. This is the case in conditions of weak surface heating, such as those encountered in stratocumulus‐topped boundary layers (Lopez‐Gomez et al., 2020). Thus, the velocity scale *w* is taken as the maximum of the previously described scales. Considerations of symmetry and mass and tracer conservation lead to the inverse time scale

(30) $$\lambda_{ij}=s_{\min}\left(\left|\frac{{\bar{b}}_{i}-{\bar{b}}_{j}}{{\bar{w}}_{i}-{\bar{w}}_{j}}\right|,c_{\lambda }\left|\frac{{\bar{b}}_{i}-{\bar{b}}_{j}}{\sqrt{{ē}_{0}}}\right|\right).$$

Here, *λ*_*ij*_ = *λ*_*ji*_, *c*_*λ*_ is a nondimensional fitting parameter, and $s_{\min}$ is the smooth minimum function defined in Lopez‐Gomez et al. (2020). The smooth minimum function ensures that the strongest characteristic velocity defines the entrainment rate. The inverse time scale *λ*_*ij*_ depends on the buoyancy difference ${\bar{b}}_{i}-{\bar{b}}_{j}$ between subdomains *i* and *j*, as is physical. Similarly, *λ*_*ij*_ depends only on the mean vertical velocity difference ${\bar{w}}_{i}-{\bar{w}}_{j}$, as is required by Galilean invariance. In terms of this inverse time scale, the entrainment and detrainment rates are then written as

(31) $$E_{ij}=\rho \lambda_{ij}\left(c_{\epsilon }D_{ij}+c_{\delta }M_{ij}\right),$$

and

(32) $$\Delta_{ij}=\rho \lambda_{ij}\left(c_{\epsilon }D_{ji}+c_{\delta }M_{ji}\right).$$

Mass and tracer conservation demand that *E*_*ij*_ = Δ_*ji*_ (see Equation 24). This is satisfied by this formulation: The inverse time scale *λ*_*ij*_ is symmetric under reversal of the *i* and *j* indices by construction. Conservation constraints are satisfied by the choice of the, as yet unspecified, nondimensional functions $D_{ij}$ and $M_{ji}$ in the entrainment rate (31) and, with inverted indices, $D_{ji}$ and $M_{ij}$ in the detrainment rate (32). The coefficients *c*_*ϵ*_ and *c*_*δ*_ are nondimensional fitting parameters. The functions $D_{ij}$ and $M_{ij}$ in principle can depend on all nondimensional groups of the problem. Once sufficient data are available, be they from high‐resolution simulations or observations, they can be learned from data.

To demonstrate the viability of the EDMF closure, we use physically motivated and relatively simple functions for $D_{ij}$ and $M_{ij}$.

#### 3.1.2 Function $D_{ij}$

We use the function $D_{ij}$ to estimate the relative magnitudes of entrainment and detrainment for a subdomain *i* in dry convection, in which case the subdomain buoyancy is linearly mixed. We consider the buoyancy ${\bar{b}}_{\text{mix}}$ of a mixture, composed of a fraction *χ*_*i*_ of air from subdomain *i*, and a fraction *χ*_*j*_ of air from subdomain *j* (with *χ*_*i*_ + *χ*_*j*_ = 1). We define an inverse time scale based on the mixture buoyancy as

(33) $$\mu_{ij}=\frac{{\bar{b}}_{\text{mix}}-{\bar{b}}_{ij}}{{\bar{w}}_{i}-{\bar{w}}_{j}},$$

where

(34) $${\bar{b}}_{ij}=\frac{a_{i}{\bar{b}}_{i}+a_{j}{\bar{b}}_{j}}{a_{i}+a_{j}}$$

is the area‐weighted mean buoyancy of subdomains *i* and *j*, such that *a*_*i*_ + *a*_*j*_ = 1 implies ${\bar{b}}_{ij}=\langle b\rangle$. (Note that we are assuming dry conditions here, so buoyancy averages linearly.) Here *μ*_*ij*_ = −*μ*_*ji*_, and its sign reflects the correlation between the sign of the velocity difference ${\bar{w}}_{i}-{\bar{w}}_{j}$ and the sign of the mixture buoyancy ${\bar{b}}_{\text{mix}}$ relative to the mean buoyancy ${\bar{b}}_{ij}$. The mixture buoyancy is defined as

(35) $${\bar{b}}_{\text{mix}}=\chi_{i}{\bar{b}}_{i}+\chi_{j}{\bar{b}}_{j},$$

so that the buoyancy difference in (33) becomes

(36) $${\bar{b}}_{\text{mix}}-{\bar{b}}_{ij}=({\bar{b}}_{i}-{\bar{b}}_{j})\left(\chi_{i}-\frac{a_{i}}{a_{i}+a_{j}}\right),$$

which follows by using *χ*_*i*_ = 1 − *χ*_*j*_.

Thus, we assumed that the more rapidly rising subdomain entrains air if the mixture buoyancy is positive relative to the mean of the two interacting subdomains, and vice versa. This means that we expect entrainment from subdomain *j* into *i* if *μ*_*ij*_ > 0, and we expect detrainment otherwise. This could be modeled by choosing $D_{ij}=\max(\mu_{ij},0)$. However, we find that using a smooth sigmoidal function, between 0 and 1, improves our results, so we define

(37) $$D_{ij}=\frac{1}{1+e^{-\mu_{ij}/\mu_{0}}}.$$

Here, *μ*_0_ is an inverse time scale, a fitting parameter that controls the smoothness of the sigmoidal function. We estimate *μ*_0_ = 4 × 10^−4^ s^−1^ from examining various LES test cases. The fact that this is a dimensional coefficient is a shortcoming of the current model; we aim to replace this by a function of grid‐mean quantities in future work. The fraction of air in the mixture, *χ*_*i*_, is typically taken from an assumed probability distribution (Bretherton et al., 2004; Kain, 2004). Here we choose a constant *χ*_*i*_ for updrafts interacting with their environment, based on a heuristic assumption of an elliptical updraft in a surrounding mixing shell. If the mixing eddies at the updraft edge have similar radial extent in the updraft and in the shell, it implies that *χ*_*i*_ is proportional to the ratio between the updraft area and the combined updraft and shell area; that is, *χ*_*i*_ = 0.25. For interactions between two updrafts (or downdrafts), the corresponding choice would be *χ*_*i*_ = *χ*_*j*_ = 0.5.

#### 3.1.3 Function $M_{ij}$

In moist conditions, the function $M_{ji}$ represents the enhancement of detrainment from the rising subdomain *i* (and entrainment into the sinking subdomain *j*) by evaporation of liquid water when *i* is cloudy (saturated). In dry conditions, we expect $M_{ji}=M_{ij}=0$. Similar to Savre and Herzog (2019), the evaporative potential of the drier subdomain *j* is approximated here by an ad hoc function of the difference between the relative humidities RH_*i*_ and RH_*j*_ of the subdomains, conditioned on the saturation of subdomain *i*:

(38) $$M_{ji}=\left\{\begin{array}{ll} {\left[\max({\bar{\text{RH}}}_{i}^{\beta }-{\bar{\text{RH}}}_{j}^{\beta },0)\right]}^{\frac{1}{\beta }}, & \text{if}\;{\bar{\text{RH}}}_{i}=1,\\ 0, & \;\text{if}{\;\bar{\text{RH}}}_{i}<1. \end{array}\right)$$

Here, *β* is a nondimensional parameter that controls the magnitude of the evaporative potential for a given relative humidity difference. With this closure, a saturated updraft *i* detrains when the environment *j* = 0 is subsaturated, and the detrainment rate increases with increasing subsaturation of the environment.

#### 3.1.4 Fractional Entrainment and Detrainment Rates

Given the relationships (28) and (29) between the entrainment rates *E*_*ij*_ and *D*_*ij*_ and their fractional counterparts *ϵ*_*ij*_ and *δ*_*ij*_, the fractional rates are

(39) $$\epsilon_{ij}=\frac{E_{ij}}{\rho a_{i}{\bar{w}}_{i}}=\frac{\lambda_{ij}}{a_{i}{\bar{w}}_{i}}\left(c_{\epsilon }D_{ij}+c_{\delta }M_{ij}\right),$$

and

(40) $$\delta_{ij}=\frac{D_{ij}}{\rho a_{i}{\bar{w}}_{i}}=\frac{\lambda_{ij}}{a_{i}{\bar{w}}_{i}}\left(c_{\epsilon }D_{ji}+c_{\delta }M_{ji}\right).$$

The relationship *E*_*ij*_ = Δ_*ji*_ required for scalar and mass conservation in terms of the fractional rates implies

$$\delta_{ji}=\frac{a_{i}{\bar{w}}_{i}}{a_{j}{\bar{w}}_{j}}\epsilon_{ij}.$$

The difference between the fractional rates, which is the source of *ρa*_*i*_, is

(41) $$\epsilon_{ij}-\delta_{ij}=\frac{\lambda_{ij}}{a_{i}{\bar{w}}_{i}}\left(c_{\epsilon }(D_{ij}-D_{ji})+c_{\delta }(M_{ij}-M_{ji})\right).$$

The function $D_{ij}-D_{ji}$ appearing here is a sigmoidal function between −1 and 1.

For the situation where entrainment is only considered between an updraft *i* and the environment *j* = 0, and if the environmental mean vertical velocity ${\bar{w}}_{0}$ and turbulent kinetic energy ${ē}_{0}$ are neglected, this closure reduces to a closure of the form ${\bar{b}}_{i}/{\bar{w}}_{i}^{2}$. It is heuristically modulated by the nondimensional functions $D_{ij}$ and $M_{ij}$, which approximate the relative magnitudes of entrainment and detrainment while accounting for enhanced detrainment owing to evaporation of condensate.

### 3.2 Turbulent Entrainment

We assume that turbulent entrainment takes place only between the plumes (updrafts and downdrafts) and their environment, where second moments are not neglected. Therefore, we assume it depends on the turbulent velocity scale of the environment, $\sqrt{{ē}_{0}}$, and the radial scale of a plume *R*_*i*_. The turbulent entrainment rate is related to the flux across the subdomain boundary via

(42) $${Ê}_{i0}({\bar{\phi }}_{0}-{\bar{\phi }}_{i})=-\rho a_{i}\frac{A_{sg}}{V_{i}}\hat{\phi^{\prime }u_{r,n}^{\prime }},$$

where *A*_*sg*_ and *V*_*i*_ are the updraft's interface area and volume (see the derivation of (B10)). We assume here that the updraft is cylindrical with a circular cross section, so that the ratio between its interface area and its volume is *A*_*sg*_/*V*_*i*_ = 2/*R*_*i*_. Following de Rooy and Siebesma (2010), Asai and Kasahara (1967), and Kuo (1962) the outward pointing turbulent flux across the boundary of the *i*th updraft, $\hat{\phi^{\prime }u_{r,n}^{\prime }}$, is modeled by downgradient eddy diffusion

(43) $$\hat{\phi \prime u\prime_{r,n}}\approx -{\hat{K}}_{i0}\frac{{\bar{\phi }}_{0}-{\bar{\phi }}_{i}}{R_{i}}=-{\hat{K}}_{i0}\;\frac{{\bar{\phi }}_{0}-{\bar{\phi }}_{i}}{\gamma H_{i}}.$$

Here ${\hat{K}}_{i0}$ is the entrainment eddy diffusivity between the environment and the *i*th subdomain. The cross‐subdomain gradient is discretized using the difference in the mean values of the two interacting subdomains and the radial scale of the updraft *R*_*i*_. The latter is written in terms of updraft height *H*_*i*_ and an aspect ratio *γ* as *R*_*i*_ = *γH*_*i*_. The updraft height *H*_*i*_ is taken to be the maximal height at which *a*_*i*_ > 0 in the previous time step, but at least 100 m to avoid division by zero in the initial stages of the simulation. For the entrainment eddy diffusivity, we assume the form

(44) $${\hat{K}}_{i0}=c_{t}R_{i}\sqrt{{ē}_{0}},$$

where *R*_*i*_ is used as a mixing length and *c*_*t*_ is a nondimensional fitting parameter.

Combining Equations 42–(44), we obtain the turbulent entrainment rate

(45) $${Ê}_{i0}=2\rho a_{i}c_{t}\frac{\sqrt{{ē}_{0}}}{R_{i}}=2\rho a_{i}c_{\gamma }\frac{\sqrt{{ē}_{0}}}{H_{i}},$$

where *c*_*γ*_ = *c*_*t*_/*γ* is a fitting parameter that combines *c*_*t*_ and *γ* (Table 2). The middle term in (45) shows that ${Ê}_{ij}\propto 1/R_{i}$, in agreement with laboratory experiments of dry plumes (Morton et al., 1956; Turner, 1963). It is also useful to define a fractional counterpart for turbulent entrainment,

(46) $${\hat{\epsilon }}_{i0}=\frac{{Ê}_{ij}}{\rho a_{i}{\bar{w}}_{i}}=\frac{2c_{\gamma }\sqrt{{ē}_{0}}}{{\bar{w}}_{i}H_{i}}.$$

**Table 2:** Closure Parameters

Symbol	Description	Value (unit)
*a*_*s*_	Combined updraft surface area fraction	0.1
*c*_*ϵ*_	Scaling constant for entrainment rate	0.13
*c*_*δ*_	Scaling constant for detrainment rate	0.52
*c*_*λ*_	Weight of TKE term in entrainment/detrainment rate	0.3
*β*	Detrainment relative humidity power law	2.0
*μ*_0_	Time scale for *b*/*w* in the entrainment sigmoidal function	4 × 10^−4^ (1/s)
*χ*_*i*_	Fraction of updraft air in buoyancy mixing	0.25
*c*_*γ*_	Scaling constant for turbulent entrainment rate	0.075

## 4 Numerical Implementation

The model equations and closures are implemented in the SCM used in Tan et al. (2018), where a detailed description of the implementation of the initial and boundary conditions is given. The model solves for first moments of the prognostic variables $\left\{a_{i},{\bar{w}}_{i},{\bar{\theta }}_{l,i},\text{and}\;{\bar{q}}_{t,i}\right\}$ in updrafts using (18), (19), and (20), respectively, and for the grid mean variables {⟨*θ*_*l*_⟩,⟨*q*_*t*_⟩} using equations of the form of (25), in which prescribed large‐scale tendencies are applied as sources.

We consider a single updraft and its turbulent environment. The mean environmental properties are computed diagnostically as the residual of updraft and grid‐mean quantities using (8) and (9). Prognostic equations for the second moments ( $\bar{\theta_{l,0}^{\prime 2}}$, $\bar{q_{t,0}^{\prime 2}}$, $\bar{\theta_{l,0}^{\prime }q_{t,0}^{\prime }}$ ${ē}_{0}$) in the environment are solved using (22) and (23). The grid‐scale second moments are diagnosed from (10), using the EDMF assumption of neglecting second moments in the updraft. Grid‐scale third moments are diagnosed using (11), neglecting third moments in all individual subdomains. Thus, from a probability density function perspective, we are using a closure model that assumes a Gaussian environment and a delta distribution updraft (Lappen & Randall, 2001a).

The parameters we use in the entrainment and detrainment closures are shown in Table 2. The parameters in this study and in Lopez‐Gomez et al. (2020) were chosen sequentially: We first calibrated a subset of parameters associated with turbulent mixing based on stable boundary layer simulations (Lopez‐Gomez et al., 2020). We then searched for a combination of parameters related to dry convection (*c*_*ϵ*_, *c*_*t*_, *c*_*λ*_) so that the EDMF scheme captures the DCBL and the subcloud layer in moist convective cases. Finally, we optimized the moisture‐related parameters (*β*,*c*_*δ*_) based on the EDMF scheme's ability to capture cloud layer properties and the cloud top height.

The initial conditions, surface fluxes, and large‐scale forcing are case specific. They are taken from the papers describing the cases, are linearly interpolated to the model resolution, and are implemented identically in the SCM and LES.

The SCM implementation of the EDMF scheme makes several assumptions because the SCM does not solve for the density, pressure, or vertical velocity of the grid‐mean. In the SCM, it is assumed that ⟨*w*⟩ = 0 and *ρ* = *ρ*_*h*_ in the EDMF equations, and consequently that *ρ* = *ρ*_*h*_ in the denominators of the buoyancy definitions (15) and (16). Furthermore, the grid‐mean anelastic approximation requires the use of the reference pressure (*p*_*h*_) in the ideal gas law (21) for consistency (Pauluis, 2008). The SCM is therefore fully anelastic, in contrast to the SGS anelastic approximation described in Appendix C. Since ⟨*w*⟩ = 0, the balance in the ⟨*w*⟩ equation is reduced to

(47) $$\langle b\rangle -\frac{1}{\rho_{h}}\frac{\partial }{\partial z}\left(\rho_{h}\langle w^{*}w^{*}\rangle \right)=\frac{\partial }{\partial z}\left(\frac{\left\langle p^{†}\right\rangle }{\rho_{h}}\right),$$

thus removing from the subdomain equations the dependence on the grid‐mean pressure.

All SCM simulations use a uniform vertical resolution of 50 m, with results from a resolution sensitivity test at 100 and 150 m shown for the first three moments in the grid. Other implementation details, such as how cloud properties are computed via numerical quadrature over implied SGS distributions, are described in Lopez‐Gomez et al. (2020).

## 5 LES and Diagnosis of EDMF Subdomains

To assess the performance of the extended EDMF scheme, we compared it with LES in four convective test cases. We use PyCLES (Pressel et al., 2015), an anelastic LES code with weighted essentially nonoscillatory (WENO) numerics. We use an implicit LES strategy, which uses the dissipation inherent to WENO schemes as the only subgrid‐scale dissipation. Such an implicit LES has been shown to outperform explicit SGS closures in simulations of low clouds (Pressel et al., 2017; Schneider et al., 2019). We use passive tracers that decay in time to diagnose updrafts and their exchanges with the environment in the LES (see Appendix D).

Four standard convective test cases are considered here: dry convective boundary layer, maritime shallow convection, continental shallow convection, and continental deep convection.

1. The Dry Convective Boundary Layer test case (DCBL, blue lines in all figures) is based on Soares et al. (2004). In this case, convection develops through 8 hr from an initially neutral profile below 1,350 m (which is stable above it) with prescribed sensible and latent heat fluxes and negligible large‐scale winds. We use an isotropic 25 m resolution in a 6.4 km × 6.4 km × 3.75 km domain.
2. The marine shallow convection test case is based on the Barbados Oceanographic and Meteorological Experiment (BOMEX, orange lines) described in Holland and Rasmusson (1973). In this case, large‐scale subsidence drying and warming and fixed surface fluxes are prescribed, and subtropical shallow cumulus convection evolves over 6 hr, with a quasi‐steady state maintained in the last 3 hr (Siebesma et al., 2003). We use an isotropic 40 m resolution in a 6.4 km × 6.4 km × 3 km domain.
3. The continental shallow convection test case is based on the Atmospheric Radiation Measurement Program at the United States's Southern Great Plains (ARM‐SGP, green lines) described in Brown et al. (2002). This case exhibits a diurnal cycle of the surface fluxes, with cumulus convection first developing and then decaying between 5:30 and 20:00 local time. We use 100 m × 100 m × 40 m resolution in a 25 km × 25 km × 4 km domain. The large surface fluxes of latent and sensible heat erode the initial inversion as convection penetrates into the free atmosphere (Brown et al., 2002).
4. The continental deep convection test case is based on the Large‐scale Biosphere‐Atmosphere experiment with data from the Tropical Rainfall Measurement Mission (TRMM‐LBA, red lines) observed on 23 February 1999 in Brazil (Grabowski et al., 2006). In this case, prescribed time‐varying surface fluxes and radiative cooling profiles force a diurnal cycle, during which shallow convection transitions into deep convection in the 6 hr between 7:30 and 13:30 local time. We use 200 m × 200 m × 50 m resolution in a 51.2 km × 51.2 km × 24 km domain. No subsidence drying or warming are prescribed in this case. In our simulations of the TRMM‐LBA case, microphysical rain processes are modeled by a simple warm‐rain cutoff scheme that removes liquid water once it is 2% supersaturated. This simple scheme is implemented in the LES for a direct comparison with the same simple microphysics scheme in SCM. In future work, we will implement a more realistic microphysics scheme.

The different cases span a wide range of conditions that allow us to examine the different components of the unified entrainment and detrainment formulation presented in section 3. The DCBL case allows us to examine the dry formulations for dynamic and turbulent entrainment irrespective of the moisture related detrainment. The differences in environmental humidity between the shallow and deep convection cases allows us to test the moisture‐dependent detrainment closure. For instance, we found the bulk detrainment value used in previous parameterization evaluated with BOMEX (Siebesma & Cuijpers, 1995; Tan et al., 2018) to be excessive for TRMM‐LBA.

The diagnosis of the direct estimates of entrainment and detrainment and comparison with the closures (39) and (40) relies on decaying tracers with a surface source, which uniquely identify each LES grid box as either updraft or environment. Here we use the tracer scheme described in Couvreux et al. (2010), which labels a grid cell as updraft if its vertical velocity, tracer concentration, and liquid water specific humidity (above cloud base) exceed given thresholds. The net of entrainment minus detrainment [right‐hand side of (18)] is diagnosed using the area and vertical velocity of updrafts identified with the help of the tracer scheme. Fractional entrainment is diagnosed based on an advective form of the scalar equation, see Equation D1. Further information on the diagnosis is found in Appendix D.

## 6 Results

**Figure 1:**
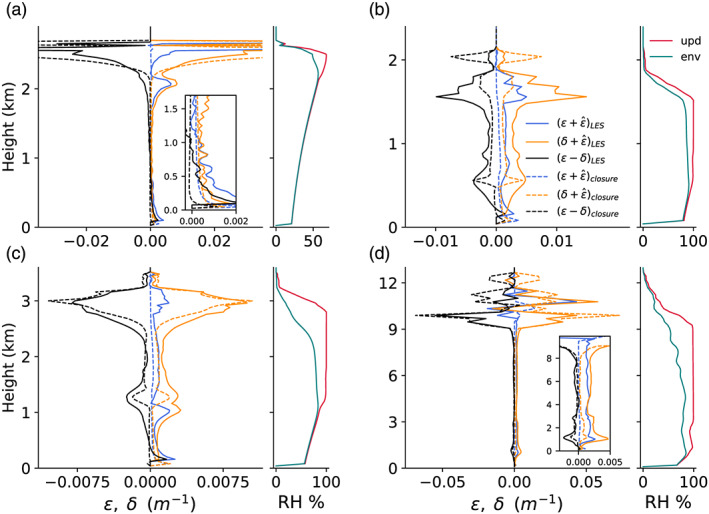
A comparison of the direct estimates (“LES,” solid lines) of fractional entrainment and detrainment rates and their closures (“closure,” dashed lines) evaluated in LES of the four convective test cases. Panels (a)–(d) show results for the DCBL, BOMEX, ARM‐SGP, and TRMM LBA test cases, respectively. For each case, the left panel shows the mean profiles of diagnosed entrainment, detrainment, and their net rate (solid lines), averaged over the last 2 hr (Hours 9–11 in ARM‐SGP), compared with the closures in (39), (40), and (41) (dashed lines). The right panel for each case shows profiles of relative humidity in the updraft (red) and environment (green). The legend in (b) applies to all panels.

A comparison of the closures for the fractional turbulent and dynamic entrainment and detrainment rates with direct estimates of these terms from LES is shown in Figure 1. In this comparison, the profiles of the EDMF closures are based on diagnosing all EDMF components (area fractions, first and second moments) from LES and using those in the EDMF closures described in section 3. The profiles of the closures for entrainment and detrainment are similar to the direct estimates from LES. The role of the environmental moisture deficit in enhancing detrainment in the cloud layer is consistent with the directly diagnosed detrainment in ARM‐SGP, in which convection penetrates into a dry layer with RH ≈ 50%.

**Figure 2:**
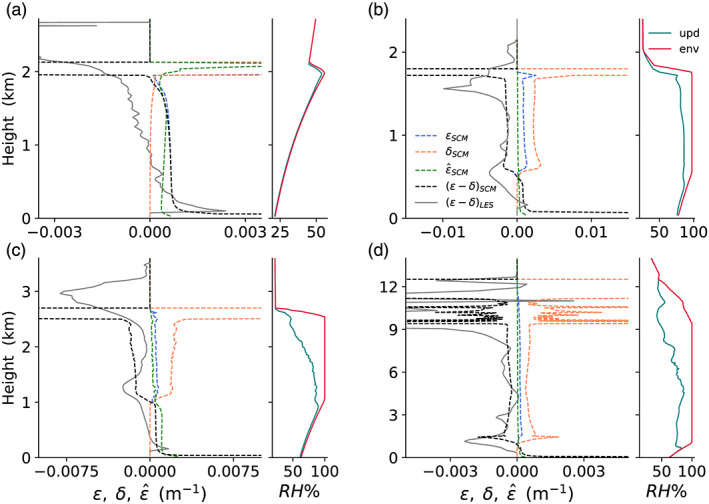
Last 2 hr mean profiles of entrainment and detrainment in the SCM simulations as in Figure 1. Dynamic entrainment rate *ϵ* (dashed‐blue), dynamic detrainment rate *δ* (dashed‐orange), net entrainment rate *ϵ* − *δ* (dashed‐black), and turbulent entrainment $\hat{\epsilon }$ (dashed‐green). The LES‐diagnosed *ϵ* − *δ*, shown in Figure 1, is added here in solid gray for comparison. The corresponding relative humidities (RH) of the updraft (red) and environment (green) are shown on the right‐hand side.

When implemented in the SCM, these closures perform in a similar manner (Figure 2). Dynamic entrainment prevails in the subcloud layer, while dynamic detrainment prevails in the cloud layer, owing to the large environmental moisture deficit. The value of *ϵ* − *δ* predicted by the closures in the EDMF scheme is in agreement with direct estimates of this value from LES (solid gray lines). Turbulent entrainment is about half the dynamic entrainment in the boundary layer and vanishes above it. A discrepancy between the SCM and LES is found between the entrainment and detrainment profiles for the DCBL case. The LES updrafts detrain from mid levels and upward, whereas the SCM updrafts detrain mostly at their tops. This could indicate of a downside of the current closure that uses the subdomain mean buoyancy and does not detrain from buoyant updrafts. A more sophisticated scheme, in which entrainment dependents on second moments, could improve the performance at the cost of computing second moment in all subdomains.

**Figure 3:**
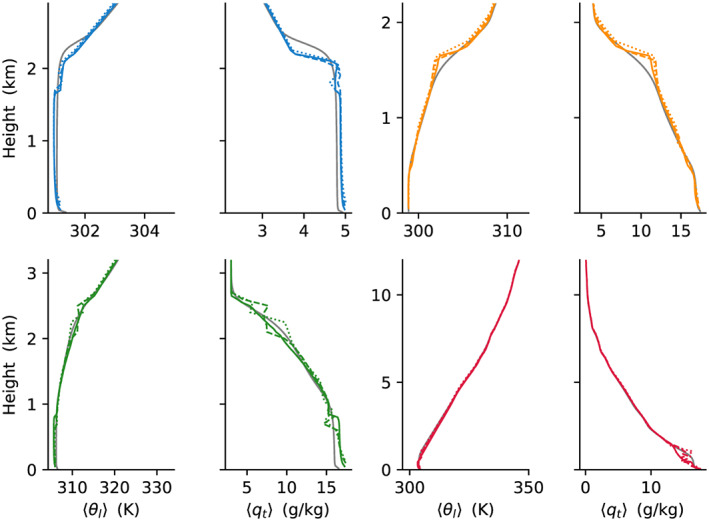
Comparison of SCM and LES for the last 2 hr (Hours 9–11 in ARM‐SGP) for mean profiles of first moments (first and third columns) ⟨*θ*_*l*_⟩ and (second and fourth columns) ⟨*q*_*t*_⟩. In all panels, color lines show SCM profiles and gray lines represent the corresponding LES profiles. DCBL, BOMEX, ARM‐SGP, and TRMM‐LBA are color‐coded as blue, orange, green, and red, respectively. Solid, dashed, and dotted color lines show SCM results for 50, 100, and 150 m resolutions, respectively.

**Figure 4:**
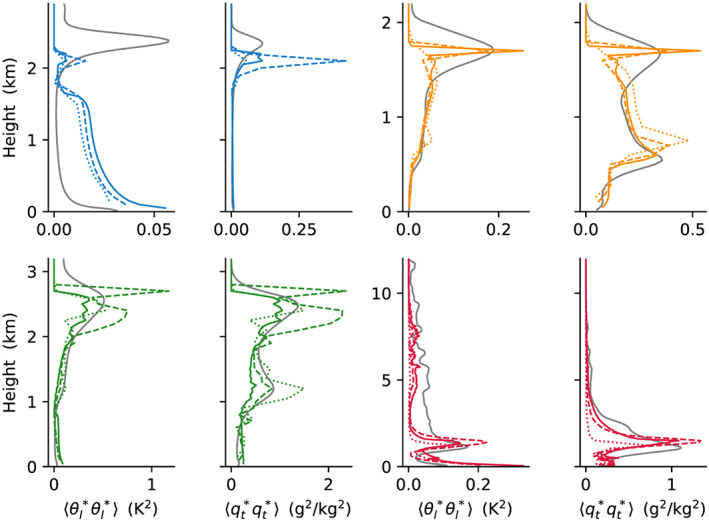
Same as Figure 3 but for the second moments: $\left\langle \theta_{l}^{*}\theta_{l}^{*}\right\rangle$ and $\left\langle q_{t}^{*}q_{t}^{*}\right\rangle$.

**Figure 5:**
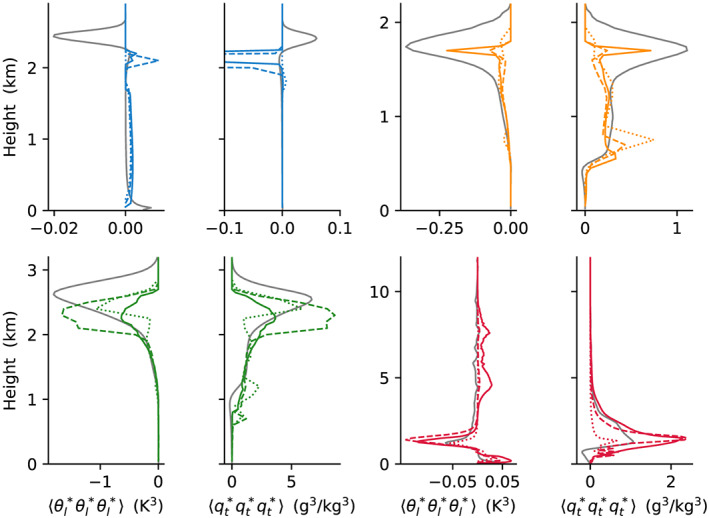
Same as Figure 3 but for the third moments $\left\langle \theta_{l}^{*}\theta_{l}^{*}\theta_{l}^{*}\right\rangle$ and $\left\langle q_{t}^{*}q_{t}^{*}q_{t}^{*}\right\rangle$. The DCBL spike in the $\left\langle q_{t}^{*}q_{t}^{*}q_{t}^{*}\right\rangle$ profile (blue) has an amplitude of −1.5 (g^3^/kg^3^).

We now turn to compare the performance of the EDMF scheme with LES. First, second, and third moments of *θ*_*l*_ and *q*_*t*_ are compared in Figures 3, 4, and 5. These show overall good matches between the SCM and LES, with a few notable mismatches. For example, in first moments in the sub‐cloud layer in the ARM‐SGP case, at cloud top in the BOMEX case, and at the top of the DCBL; in second moments ( $\left\langle \theta_{l}^{{*}^{2}}\right\rangle$) throughout the DCBL; and in the third moments at the overshoots. Moreover, mismatches in sign are seen for $\left\langle \theta_{l}^{{*}^{3}}\right\rangle$ in SCM simulations of TRMM‐LBA at mid levels, and for $\left\langle q_{t}^{{*}^{3}}\right\rangle$ at the top of the DCBL. The sensitivity test at 100 (dashed color lines) and 150 m (dotted color lines) resolution in these figures shows that these results are generally robust to the vertical resolutions expected in the host model.

**Figure 6:**
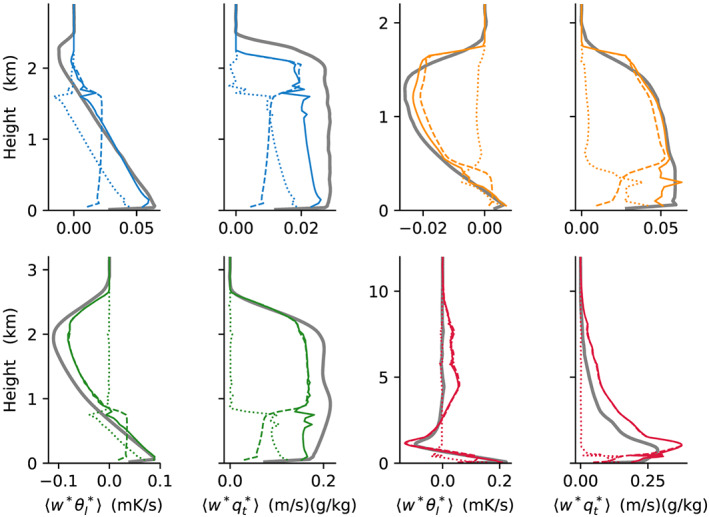
Solid lines show a comparison of the vertical fluxes $\left\langle w^{*}\theta_{l}^{*}\right\rangle$ and $\left\langle w^{*}q_{t}^{*}\right\rangle$ in the grid with similar color coding of as in Figure 3. Dotted and dashed lines show in addition the SCM diffusive flux (ED) and massflux (MF) components, respectively. The SCM vertical resolution in this figure is 50 m.

The grid‐mean SGS fluxes, whose divergence is a source in the host model equations, are shown in Figure 6. We find good agreement in the fluxes except for $\left\langle w^{*}\theta_{l}^{*}\right\rangle$ in TRMM‐LBA at midlevels, where the SCM shows a strongly positive flux while the LES has a negligible flux there. The ED and MF components of the SCM fluxes show that the ED components (dotted) are limited to the boundary layer where ${ē}_{0}$ is nonnegligible and the MF component (dashed) dominates above it, as expected.

**Figure 7:**
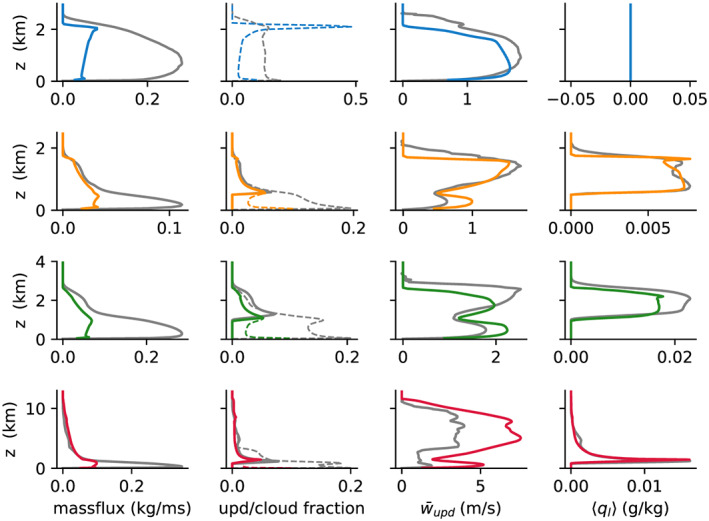
Mean profiles of cloud properties over the last 2 hr (Hours 9–11 in ARM‐SGP). Top to bottom rows correspond to DCBL, BOMEX, ARM‐SGP. and TRMM‐LBA, with SCM following the color‐coding in Figure 3 and corresponding LES in gray. Left to right columns correspond to updraft massflux, updraft fraction (dashed) and cloud fraction (solid), updraft vertical velocity, and liquid water specific humidity, respectively.

The comparison of updraft and cloud properties in Figure 7 shows good agreement with LES above cloud base. Below cloud base and in the DCBL, large disagreements in the mass flux and updraft fractions are found. However, in the boundary layer, the diagnosis of updrafts in the LES can be misleading because lateral turbulent mixing makes the distinction between updrafts and their environment ambiguous. We did not attempt to implement a more sophisticated scheme, such as (Efstathiou et al., 2020) in this work. However, the key predictions of the EDMF scheme (the SGS vertical fluxes and the mean profiles on the host model grid) are in good agreement with the LES (Figure 6). This implies that the net of ED and MF effects in the SCM reproduces the well‐mixed boundary layer, even though the decomposition into updrafts and environment may not be exact.

**Figure 8:**
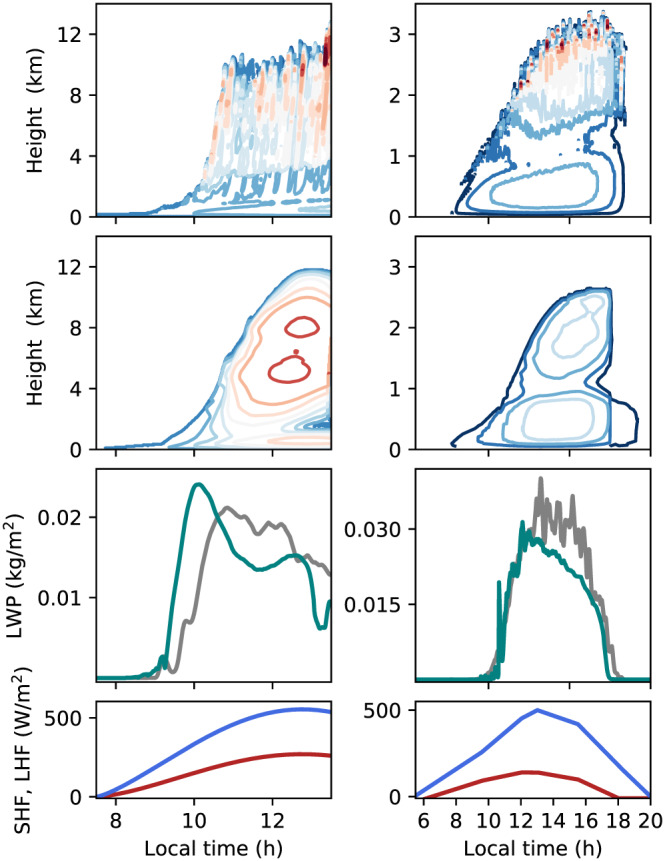
Diurnal cycle in the TRMM‐LBA case (left column) and in the ARM‐SGP case (right column). Contours show updraft vertical velocity in the LES (first row) and SCM (second row). Contour levels are at (− 2, − 1, … ,10) m *s*^−*1*^ for TRMM‐LBA and at (0.5,0, … ,4.5) m *s*^−*1*^ for ARM‐SGP. The third row shows the liquid water path (LWP) in the SCM (green) and LES (gray). The bottom row shows the surface latent flux (blue) and sensible heat flux (red).

**Figure 9:**
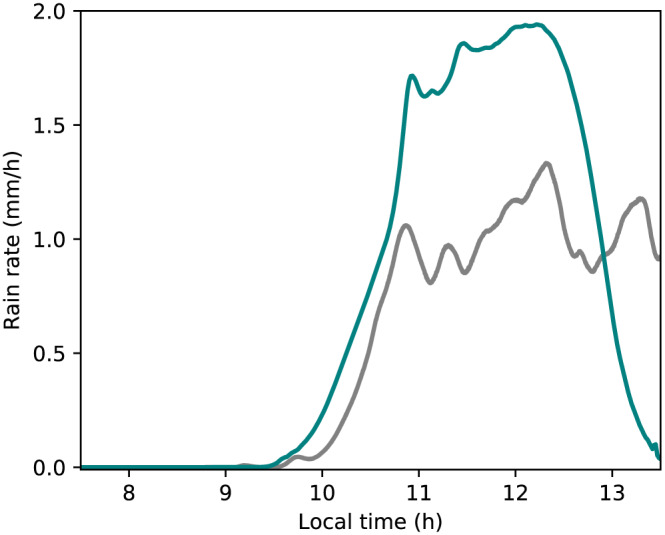
A comparison of the rain rates in the TRMM‐LBA case between the SCM (green) and LES (gray).

Diurnal cycles of shallow and deep convection are shown in Figure 8. The onset of convection in the SCM is found to be about half an hour delayed compared with the LES, while cloud top height is in good agreement between the models. In the decay stage in the ARM‐SGP case, the cloud in the SCM shuts off abruptly, unlike the gradual decline in the LES. This may result from the EDMF assumption that neglects variance in the (single) updraft, which cannot cross cloud base when its buoyancy right below cloud base is too low. Good agreement is found in the liquid water path (LWP) between the SCM and the LES in both cases. In the TRMM‐LBA case, this agreement includes the effect of precipitation on the column integrated *q*_*t*_. The precipitation sink is used to compute rain rates in the cutoff microphysics scheme as the vertically integrated amount of *q*_*t*_ removed at a model time step per unit area. The EDMF rain rates peak at nearly twice their LES counterparts in the TRMM‐LBA case (Figure 9). This overestimation is consistent with the overestimation of ${\bar{w}}_{upd}$ (Figure 7). Tuning the maximum supersaturation in the cutoff microphysics could improve both the vertical velocity and the rain rates, although this was not explored here. The coarse‐graining of the convective plumes into a single updraft in the EDMF scheme may indicate that a different supersaturation should be applied in the SCM compared with the LES.

## 7 Discussions and Conclusions

We have presented entrainment and detrainment closures that allow the extended EDMF scheme to simulate boundary layer turbulence, shallow convection, and deep convection, all within a unified physical framework. The results demonstrate the potential of the extended EDMF scheme to serve as a unified parameterization for all SGS turbulent and convective motions in climate models (other SGS motions such as gravity waves require additional parameterizations). The choice of parameters used to produce these results is uniform across all cases, as well as across all cases shown in Lopez‐Gomez et al. (2020). We view these results as a proof of concept, which we will improve further using automated model calibration techniques and a larger LES data set in the future.

The dynamic entrainment/detrainment closures are based on a combination of a *b*/*w*^2^ scaling and physically motivated nondimensional functions, which can in principle be learnt from data. At the moment, these functions are based on arguments from buoyancy sorting and relative humidity differences between clouds and their environment. The addition of turbulent entrainment, which only affects scalars, allows us to regulate the mass flux by reducing the vertical velocity without increasing the area fraction below cloud base, where detrainment is negligible.

The extended EDMF scheme produces good agreement with LES in key properties needed for climate modeling. The successful simulation of high‐order moments and vertical fluxes justifies the EDMF assumption of a negligible contribution from updraft covariance to the grid scale covariance. It would be straightforward to include multiple updrafts (Neggers, 2012; Neggers et al., 2002; Sušelj et al., 2012), which can further improve the results. Using multiple updrafts would also open up the opportunity to include stochastic components either in the updrafts' boundary conditions or in the entrainment and detrainment closures (Romps, 2016; Suselj et al., 2013, 2014, 2019a), with the nonlinearity of the model ensuring that the stochastic effect will not average out in the grid mean. Nonetheless, the use of multiple updrafts results in a higher computational overhead of the parameterization in climate simulations. This added cost may be ameliorated harnessing the power of parallel architectures.

There is a growing interest in using artificial neural networks as SGS models for turbulence and convection (e.g., O'Gorman & Dwyer, 2018; Rasp et al., 2018). It is worth noting that the extended EDMF scheme with multiple updraft and downdraft has a network structure: The subdomains play the role of network nodes, which interact through sigmoidal activation functions (entrainment/detrainment). Each node has memory (explicitly time‐dependent terms), somewhat akin to long short‐term memory (LSTM) networks (Hochreiter & Schmidhuber, 1997). Unlike artificial neural networks whose architecture is not tailor‐made for the physical problem at hand, the architecture of the extended EDMF scheme ensures physical realizability and conservation of energy. Like for neural networks, the activation functions and other parameters in the extended EDMF scheme can be learnt from data. Our results, which required adjustment of only a handful of parameters, show that only a small fraction of the data typically required to train neural networks is needed to calibrate the extended EDMF scheme.

The explicitly time‐dependent nature of the extended EDMF scheme makes it well suited to operate across a wide range of GCM resolutions and under time‐varying large‐scale conditions that may include diurnal cycles and variability on even shorter time scales (Tan et al., 2018).

## Data Availability Statements

The PyCLES code used to generate LES results is available at this site (climate-dynamics.org/software/#pycles). The SCM code is available at this site (https://doi.org/10.5281/zenodo.3789011).

## Appendix A Computation of Central Second and Third Moments

### A.1

The second moment of SGS variations is given in terms of the EDMF decomposition by applying the Reynolds decomposition to the product of two scalars,

(A1) $$\langle \phi^{*}\psi^{*}\rangle =\langle \phi \psi \rangle -\langle \phi \rangle \langle \psi \rangle ,$$

and applying the subdomain decomposition to the first term on the right‐hand side of (A1):

(A2) $$\langle \phi^{*}\psi^{*}\rangle =\underset{i\geq 0}{\sum }a_{i}\bar{\phi_{i}^{\prime }\psi_{i}^{\prime }}+\underset{i\geq 0}{\sum }a_{i}{\bar{\phi }}_{i}{\bar{\psi }}_{i}-\langle \phi \rangle \langle \psi \rangle .$$

Multiplying the last term on the right‐hand side of (A2) by (8) (which equals unity), the entire right‐hand side of this equation yields the first equality in (10). Alternatively, replacing the grid‐mean scalars ⟨*ψ*⟩ and ⟨*ϕ*⟩ in (A2) by (9) and combining the summations of mean terms yields

(A3) $$\langle \phi^{*}\psi^{*}\rangle =\underset{i\geq 0}{\sum }a_{i}\bar{\phi_{i}^{\prime }\psi_{i}^{\prime }}+\underset{i\geq 0}{\sum }\underset{j\geq 0}{\sum }a_{i}a_{j}{\bar{\phi }}_{i}({\bar{\psi }}_{i}-{\bar{\psi }}_{j}).$$

From here, the second equality in (10) is derived by splitting the second summation in (A3) into two identical terms with a factor 1/2, replacing the role of *i* and *j* in one of them and summing them back together.

Similarly, the third moment of SGS variations is given by considering the product of three scalars as a single variable,

(A4) $$\langle \phi \psi w\rangle =\underset{i\geq 0}{\sum }a_{i}{\left(\bar{\phi \psi w}\right)}_{i}.$$

The mean product of three joint scalars can be decomposed as

(A5) $$\langle \phi \psi w\rangle =\langle \phi^{*}\psi^{*}w^{*}\rangle +\langle \phi \rangle \langle \psi^{*}w^{*}\rangle +\langle \psi \rangle \langle \phi^{*}w^{*}\rangle +\langle w\rangle \langle \psi^{*}\phi^{*}\rangle +\langle \phi \rangle \langle \psi \rangle \langle w\rangle ,$$

and in the *i*th subdomain it is

(A6) $${\left(\bar{\phi \psi w}\right)}_{i}=\bar{\phi_{i}^{\prime }\psi_{i}^{\prime }w_{i}^{\prime }}+{\bar{\phi }}_{i}\bar{\psi_{i}^{\prime }w_{i}^{\prime }}+{\bar{\psi }}_{i}\bar{\phi_{i}^{\prime }w_{i}^{\prime }}+{\bar{w}}_{i}\bar{\psi_{i}^{\prime }\phi_{i}^{\prime }}+{\bar{\phi }}_{i}{\bar{\psi }}_{i}{\bar{w}}_{i}.$$

Substituting (A5) and (A6) into (A4) yields (11). Finally, in LES diagnosis the centered third moment is computed using the domain averages of the scalar, its square, and its cube as

(A7) $$\langle \phi^{*}\phi^{*}\phi^{*}\rangle =\langle {\left(\phi -\langle \phi \rangle \right)}^{3}\rangle =\langle \phi^{3}\rangle -3\langle \phi \rangle \langle \phi \phi \rangle +2{\left\langle \phi \right\rangle }^{3}.$$

## Appendix B Derivation of Subdomain First and Second Moment Equations

### B.1

Here we derive the prognostic equations for the subdomain area fraction *a*_*i*_, the subdomain‐mean, and the subdomain covariance for any pair of scalars *ϕ*, *ψ*. In this derivation, we assume *ρ*_*i*_ = ⟨*ρ*⟩ anywhere but in the buoyancy term, much like in the anelastic model. This “SGS anelastic” assumption removes subgrid‐scale sound waves and circumvents the need to define a subdomain pressure (Thuburn et al., 2019). The molecular viscosity and diffusivity are both neglected in the first moment equations, but are reintroduced in the second moment equations in order to account for the dissipation of covariance at the smallest scales.

The subdomain‐averaged equations are derived by averaging the governing equations in flux form over the subdomain Ω_*i*_. For scalar *ϕ*:

(B1) $$\int_{\Omega_{i}(t)}\frac{\partial \rho \phi }{\partial t}dV+\int_{\Omega_{i}(t)}\nabla \cdot (\rho \phi u)dV=\int_{\Omega_{i}(t)}\rho S_{\phi }dV.$$

Without loss of generality, the subdomain boundary *∂*Ω_*i*_ can be expressed as the union $\partial \Omega_{i}=\partial \Omega_{i}^{g}\cup \partial \Omega_{i}^{sg}$, where $\partial \Omega_{i}^{g}=\partial \Omega_{i}\cap \partial \Omega_{T}$ is the part of the subdomain Ω_*i*_ boundary that coincides with the grid box Ω_*T*_ boundary. The domain and subdomain boundaries are related through $\sum_{i}\partial \Omega_{i}^{g}=\Omega_{T}$. The subgrid boundary $\partial \Omega_{i}^{sg}$ is a free‐moving surface with velocity **u**_*b*_, while boundary $\partial \Omega_{i}^{g}$ is fixed. Using the Reynolds transport theorem for the transient term, the Gauss‐Ostrogradsky theorem for the divergence, and rearranging the surface integrals yields

(B2) $$\frac{\partial }{\partial t}\int_{\Omega_{i}(t)}\rho \phi dV+\int_{\partial \Omega_{i}^{g}}\rho \phi u\cdot ndS=-\int_{\partial \Omega_{i}^{sg}(t)}\rho \phi (u-u_{b})\cdot ndS+\int_{\Omega_{i}(t)}\rho S_{\phi }dV,$$

where **n** is the outward pointing unit vector normal to the surface over which the integration is performed. The first term on the right‐hand side is the flux out of subdomain Ω_*i*_ into other subdomains within the same grid box, and the second term on the left‐hand side is the flux out of subdomain Ω_*i*_ to a neighboring grid box. The total grid‐scale divergence equals the sum of fluxes from all subdomains across the grid box,

(B3) $$\nabla \cdot \int_{\Omega_{T}}(\rho \phi u)dV=\int_{\Omega_{T}}\nabla \cdot (\rho \phi u)dV=\underset{i\geq 0}{\sum }\int_{\partial \Omega_{i}^{g}}\rho \phi u\cdot ndS,$$

where the commutativity of the gradient and the volume average is exact for uniform grids and results in a small error otherwise (Fureby & Tabor, 1997). Using the domain decomposition in (9), the leftmost term in (B3) can be written in terms of the sum of the subdomain‐mean values,

(B4) $$\underset{i\geq 0}{\sum }\nabla \cdot [\rho V_{i}{\left(\bar{\phi u}\right)}_{i}]=\underset{i\geq 0}{\sum }\int_{\partial \Omega_{i}^{g}}\rho \phi u\cdot ndS,$$

where *V*_*i*_ is the volume of subdomain Ω_*i*_, and (B4) holds generally. Note that the divergence in (B4) acts on the grid scale. The diagnosis of the contribution of each subdomain to the grid‐mean divergence requires an assumption regarding the fraction of *∂*Ω_*T*_ covered by each $\partial \Omega_{i}^{g}$. Here, we assume that $A_{i}^{g}=a_{i}A_{T}^{g}$, where $A_{i}^{g}$ and $A_{T}^{g}$ are the areas of surfaces $\partial \Omega_{i}^{g}$ and *∂*Ω_*T*_, respectively. We further assume that for each Ω_*i*_ the average over $\partial \Omega_{i}^{g}$ equals the subdomain mean. From this it follows that

(B5) $$\int_{\partial \Omega_{i}^{g}}\rho \phi u\cdot ndS=\nabla \cdot [\rho V_{i}{\left(\bar{\phi u}\right)}_{i}]=\nabla \cdot [\rho V_{i}({\bar{\phi }}_{i}{\bar{u}}_{i}+\bar{\phi_{i}^{\prime }u_{i}^{\prime }})].$$

Note that (B5) cannot be obtained from the divergence theorem, since $\partial \Omega_{i}^{g}$ is not a closed surface. Using (B5) and dividing by the grid box volume *V*_*T*_, we can rewrite (B2) as

(B6) $$\frac{\partial (\rho a_{i}{\bar{\phi }}_{i})}{\partial t}=-\nabla \cdot [\rho a_{i}({\bar{\phi }}_{i}{\bar{u}}_{i}+\bar{\phi_{i}^{\prime }u_{i}^{\prime }})]-\frac{1}{V_{T}}\int_{\partial \Omega_{i}^{sg}(t)}\rho \phi u_{r}\cdot ndS+\rho a_{i}\bar{S_{\phi }},$$

where **u**_*r*_ = **u** − **u**_*b*_. Since the vertical extent of the volumes is fixed at the model vertical resolution, *V*_*i*_/*V*_*T*_ = ⟨*A*_*i*_⟩/*A*_*T*_ = *a*_*i*_, with *a*_*i*_ as the area fraction.

The net entrainment flux can be written in terms of a contribution from net mass entrainment and a contribution due to the subfilter‐scale flux of *ϕ*:

(B7) $$\frac{1}{V_{T}}\int_{\partial \Omega_{i}^{sg}(t)}\rho \phi u_{r}\cdot ndS=\frac{A_{sg}}{V_{T}}(\underset{\text{dynamical}}{\underbrace{\rho \hat{\phi }\hat{u_{r,n}}}}+\underset{\text{turbulent}}{\underbrace{\rho \hat{\phi \prime u\prime_{r,n}}}}).$$

Here, $\hat{\left(\cdot \right)}$ represents the average over interface $\partial \Omega_{i}^{sg}$, *u*_*r*,*n*_ = **u**_*r*_ · **n**, and *A*_*sg*_ is the total area of surface $\partial \Omega_{i}^{sg}$. The two terms on the right‐hand side of (B7) are denoted as net dynamical and turbulent entrainment fluxes, respectively. The net dynamical entrainment flux is taken to be the sum of two terms. For mass, it is written as

(B8) $$-\frac{A_{sg}}{V_{T}}(\rho \hat{u_{r,n}})=\underset{j\neq i}{\sum }(E_{ij}-\Delta_{ij}),$$

and for a scalar as

(B9) $$-\frac{A_{sg}}{V_{T}}(\rho \hat{\phi }\hat{u_{r,n}})=\underset{j\neq i}{\sum }(E_{ij}{\bar{\phi }}_{j}-\Delta_{ij}{\bar{\phi }}_{i}),$$

where the entrainment *E*_*ij*_ and the detrainment Δ_*ij*_ are positive semidefinite. We make the upwind approximation that the exchanged air mass carries with it the property of the subdomain from which it emanates, as is common in parameterizations (de Rooy et al., 2013).

The turbulent entrainment flux does not involve mass exchange between subdomains, and it is modeled as shown in section 3.2:

(B10) $$-\frac{A_{sg}}{V_{T}}(\rho \hat{\phi^{\prime }u_{r,n}^{\prime }})=\underset{j\neq i}{\sum }{Ê}_{ij}({\bar{\phi }}_{j}-{\bar{\phi }}_{i}).$$

Here, ${Ê}_{ij}$ is the turbulent entrainment rate from the *j*th subdomain into the *i*th subdomain. Using (B9) and (B10), decomposing the divergence term into vertical and horizontal components, and applying the eddy diffusivity assumption for the vertical turbulent flux, (B6) is written in the form (20). By setting *ϕ* = 1 in (20), the mass continuity (i.e., area fraction) Equation (18) follows.

The second‐moment equations can be derived by first writing (B6) for the product of two scalars *ϕ**ψ*. Using (B7), and decomposing the divergence term into vertical and horizontal components, we obtain

(B11) $$\begin{array}{ccc} \frac{\partial (\rho a_{i}\bar{\phi_{i}\psi_{i}})}{\partial t}+\nabla_{h}\cdot (\rho a_{i}\langle u_{h}\rangle \bar{\phi_{i}\psi_{i}}) & +\frac{\partial (\rho a_{i}\bar{\phi_{i}\psi_{i}}{\bar{w}}_{i})}{\partial z}+\frac{\partial (\rho a_{i}\bar{{\left(\phi_{i}\psi_{i}\right)}^{\prime }w_{i}^{\prime }})}{\partial z}= & \\ & -\frac{A_{sg}}{V_{T}}\left(\rho \hat{\phi \psi }\hat{u_{r,n}}+\rho \hat{\left(\phi \psi \right)\prime u_{r,n}^{\prime }}\right)+\rho a_{i}\bar{S_{\phi \psi ,i}}. \end{array}$$

The subdomain covariance equation can then be obtained from (20), (18), and (B11) as

(B12) $$\frac{\partial (\rho a_{i}\bar{\phi_{i}^{\prime }\psi_{i}^{\prime }})}{\partial t}=\frac{\partial (\rho a_{i}\bar{\phi_{i}\psi_{i}})}{\partial t}-{\bar{\psi }}_{i}\frac{\partial (\rho a_{i}{\bar{\phi }}_{i})}{\partial t}-{\bar{\phi }}_{i}\frac{\partial (\rho a_{i}{\bar{\psi }}_{i})}{\partial t}+{\bar{\phi }}_{i}{\bar{\psi }}_{i}\frac{\partial (\rho a_{i})}{\partial t},$$

which leads to

(B13) $$\begin{array}{ccc} \frac{\partial (\rho a_{i}\bar{\phi_{i}^{\prime }\psi_{i}^{\prime }})}{\partial t} & +\nabla_{h}\cdot (\rho a_{i}\langle u_{h}\rangle \bar{\phi_{i}^{\prime }\psi_{i}^{\prime }}))+\frac{\partial (\rho a_{i}{\bar{w}}_{i}\bar{\phi_{i}^{\prime }\psi_{i}^{\prime }})}{\partial z}= & \\ \frac{\partial (\rho a_{i}\bar{w_{i}^{\prime }\phi_{i}^{\prime }\psi_{i}^{\prime }})}{\partial z} & -\rho a_{i}\bar{w_{i}^{\prime }\phi_{i}^{\prime }}\frac{\partial \bar{\psi_{i}}}{\partial z}-\rho a_{i}\bar{w_{i}^{\prime }\psi_{i}^{\prime }}\frac{\partial \bar{\phi_{i}}}{\partial z}-\rho \frac{A_{sg}}{V_{T}}\left(\hat{\phi^{\prime }\psi^{\prime }{u^{\prime }}_{r,n}}-({\bar{\psi }}_{i}-\hat{\psi })\hat{u_{r,n}^{\prime }\phi^{\prime }}-({\bar{\phi }}_{i}-\hat{\phi })\hat{u_{r,n}^{\prime }\psi^{\prime }}\right)\\ & -\rho \frac{A_{sg}}{V_{T}}\left({û}_{r,n}(\hat{\phi }-{\bar{\phi }}_{i})(\hat{\psi }-{\bar{\psi }}_{i})+{û}_{r,n}\hat{\phi^{\prime }\psi^{\prime }}\right)-\rho a_{i}\bar{D_{\phi^{\prime }\psi^{\prime },i}}+\rho a_{i}(\bar{S_{\phi ,i}^{\prime }\psi^{\prime }}+\bar{S_{\psi ,i}^{\prime }\phi^{\prime }})]. \end{array}$$

Here, terms of the form $({\bar{\psi }}_{i}-\hat{\psi })\hat{u_{r,n}^{\prime }\phi^{\prime }}$ are written as ${\bar{\psi }}_{i}^{*}\hat{u_{r,n}^{\prime }\phi^{\prime }}$ to ensure conservation of second moments on the host model grid. The last term in (B13) follows from (B12), given that

(B14) $$\bar{S_{\phi \psi ,i}}=\bar{\phi_{i}S_{\psi ,i}}+\bar{\psi_{i}S_{\phi ,i}}.$$

The dissipation of covariance is represented by $\bar{D_{\phi^{\prime }\psi^{\prime },i}}$. The vertical subgrid covariance flux is written as downgradient and proportional to the eddy diffusivity *K*_*ϕ**ψ*,*i*_:

(B15) $$\frac{\partial (\rho a_{i}\bar{w_{i}^{\prime }\phi_{i}^{\prime }\psi_{i}^{\prime }})}{\partial z}=-\frac{\partial }{\partial z}\left[\rho a_{i}K_{\phi \psi ,i}\frac{\partial }{\partial z}\left(\bar{\phi_{i}^{\prime }\psi_{i}^{\prime }}\right)\right].$$

Substituting (B9), (B10), and (B15) in (B13), we obtain (22). The extended EDMF scheme only makes use of covariance equations for thermodynamic variables *θ*_*l*_ and *q*_*t*_ and for the turbulence kinetic energy. Subgrid‐scale covariances between thermodynamic variable and momentum are modeled diffusively following (14).

## Appendix C Energy Conserving Form of the SGS Anelastic Approximation

### C.1

The SGS anelastic approximation amounts to assuming ${\bar{\rho }}_{i}=\langle \rho \rangle$ everywhere except in the gravity term in the vertical momentum equation. Following Pauluis (2008), the energy‐conserving form for the SGS anelastic approximation can be derived from a linear expansion of the density about its grid‐mean value, considering independently the changes with respect to pressure and with respect to temperature and humidity. Linearizing the density about ⟨*ρ*⟩, we write

(C1) $${\bar{\rho }}_{i}({\bar{\theta }}_{l,i},{\bar{q}}_{t,i},{\bar{p}}_{i})=\langle \rho \rangle +\delta {\bar{\rho }}_{i}({\bar{\theta }}_{l,i},{\bar{q}}_{t,i},\langle p\rangle )+{\left(\frac{\partial \rho }{\partial p}\right)}_{\theta_{l},q_{t}}({\bar{p}}_{i}-\langle p\rangle ).$$

Substituting (C1) in (15), the subdomain buoyancy is written as

(C2) $${\bar{b}}_{i}=\underset{\approx {\bar{b}}_{i}}{\underbrace{-g\frac{\delta {\bar{\rho }}_{i}+\langle \rho \rangle -\rho_{h}}{\left\langle \rho \right\rangle }}}-\underset{\text{SGS sound‐waves}}{\underbrace{\frac{g}{\left\langle \rho \right\rangle }{\left(\frac{\partial \rho }{\partial p}\right)}_{\theta_{l},q_{t}}({\bar{p}}_{i}-\langle p\rangle )}}.$$

By using the first term on the right‐hand side as the effective subdomain buoyancy, the SGS sound waves represented by the second term are neglected. The subdomain perturbation pressure gradient is written using the SGS anelastic approximation as

(C3) $$-\frac{1}{\left\langle \rho \right\rangle }\frac{\partial {\bar{p}}_{i}^{†}}{\partial z}=-\frac{\partial }{\partial z}\left(\frac{{\bar{p}}_{i}^{†}}{\left\langle \rho \right\rangle }\right)-\frac{{\bar{p}}_{i}^{†}}{{\left\langle \rho \right\rangle }^{2}}\frac{\partial \langle \rho \rangle }{\partial z}=-\frac{\partial }{\partial z}\left(\frac{{\bar{p}}_{i}^{†}}{\left\langle \rho \right\rangle }\right)-\frac{{\bar{p}}_{i}^{†}}{{\left\langle \rho \right\rangle }^{2}}\frac{\partial \langle \rho \rangle }{\partial p_{h}}\frac{\partial p_{h}}{\partial z}.$$

An energy conserving form of this “SGS anelastic” approximation (i.e., with ⟨*ρ*⟩ inside the pressure gradient term) is obtained by a mutual cancelation between the last terms on the right‐hand sides of (C3) and (C2). This cancelation of terms is obtained by applying the hydrostatic balance and assuming

$$\frac{{\bar{p}}_{i}-\langle p\rangle }{\rho_{h}}{\left(\frac{\partial \rho }{\partial p}\right)}_{\theta_{l},qt}\approx \frac{{\bar{p}}_{i}-p_{h}}{\left\langle \rho \right\rangle }\frac{\partial \langle \rho \rangle }{\partial p_{h}}.$$

This derivation differs from that in Pauluis (2008) by the fact that the grid‐mean values are not necessarily hydrostatic. By setting the grid‐mean value to the reference value for both pressure and density, equation (6) in Pauluis (2008) is recovered. Using these assumptions in the subdomain vertical velocity equation provides the justification for the energy conserving form of the pressure term in (19).

## Appendix D Entrainment and Detrainment Diagnosis From LES

### D.1

The direct estimation of entrainment and detrainment is based on calculating *ϵ* − *δ* from (18), while $\epsilon +\hat{\epsilon }$ can be independently estimated from the advective form of the equation for ${\bar{q}}_{t,i}$,

(D1) $$\frac{\partial {\bar{q}}_{t,i}}{\partial t}+{\bar{w}}_{i}\frac{\partial {\bar{q}}_{t,i}}{\partial z}+\frac{1}{\rho a_{i}}\frac{\partial (\rho a_{i}\bar{w_{i}^{\prime }q_{t,i}^{\prime }})}{\partial z}={\bar{w}}_{i}\underset{j\neq i}{\sum }(\epsilon_{ij}+{\hat{\epsilon }}_{ij})({\bar{q}}_{t,j}-{\bar{q}}_{t,i})+S_{{\bar{q}}_{t,i}}.$$

When considering the decomposition into one updraft and its environment, this reduces to

(D2) $$\epsilon_{i0}+{\hat{\epsilon }}_{i0}=\frac{1}{{\bar{w}}_{i}({\bar{q}}_{t,0}-{\bar{q}}_{t,i})}\left(\frac{\partial {\bar{q}}_{t,i}}{\partial t}+{\bar{w}}_{i}\frac{\partial {\bar{q}}_{t,i}}{\partial z}+\frac{1}{{\bar{\rho }}_{i}a_{i}}\frac{\partial ({\bar{\rho }}_{i}a_{i}\bar{w_{i}^{\prime }q_{t,i}^{\prime }})}{\partial z}-S_{{\bar{q}}_{t,i}}\right).$$

Note that the vertical turbulent flux is added in this diagnostic equation for the updrafts, even though it is neglected in updrafts in the EDMF scheme. It was found that without this vertical turbulent flux in the diagnosis, the estimated *ϵ*_*i*0_ is much more likely to result in unphysical (i.e., negative) values.

## Appendix E Derivation of Entrainment Function From Conditions on the Mass Flux and Velocity Ratio at Cloud Top

### E.1

The vertical mass flux is defined as $\rho a_{i}{\bar{w}}_{i}$. As $z\to z_{\text{top}}$, the height at which the area fraction vanishes, the ratio between the mass flux and the vertical velocity should be maintained:

(E1) $$\underset{z\to z_{\text{top}}}{\lim}\left[\frac{\rho a_{i}{\bar{w}}_{i}}{{\bar{w}}_{i}}\right]=\underset{z\to z_{\text{top}}}{\lim}\left[\frac{\partial (\rho a_{i}{\bar{w}}_{i})/\partial z}{\partial {\bar{w}}_{i}/\partial z}\right]=\rho a_{i}.$$

Here, we used L'Hopital's rule. Using the steady form of (18) in the numerator and the advective form of (19) in the denominator, we obtain

(E2) $$\underset{z\to z_{\text{top}}}{\lim}\left[\frac{\rho a_{i}{\bar{w}}_{i}(\epsilon_{i0}-\delta_{i0})}{\left[{\bar{b}}_{i}-\partial ({\bar{p}}_{i}^{†}/\rho )/\partial z]/{\bar{w}}_{i}-(\epsilon_{i0}+{\hat{\epsilon }}_{i0})({\bar{w}}_{i}-{\bar{w}}_{0}\right)}\right]=\rho a_{i},$$

where the turbulent transport inside the updraft has been neglected. This equation implies

(E3) $$\delta_{i0}=\epsilon_{i0}\left(2-\frac{{\bar{w}}_{0}}{{\bar{w}}_{i}}\right)+{\hat{\epsilon }}_{i0}\left(1-\frac{{\bar{w}}_{0}}{{\bar{w}}_{i}}\right)-\frac{1}{{\bar{w}}_{i}^{2}}\left[{\bar{b}}_{i}-\frac{\partial }{\partial z}\left(\frac{{\bar{p}}_{i}^{†}}{\rho }\right)\right].$$

If we further assume that in this limit, $\epsilon +\hat{\epsilon }\ll \delta$, the above equation provides a functional form for *δ* similar to that obtained by Romps (2016).
